# Lateral motor column axons execute a ternary trajectory choice between limb and body tissues

**DOI:** 10.1186/1749-8104-2-13

**Published:** 2007-07-02

**Authors:** Victor Luria, Ed Laufer

**Affiliations:** 1 Department of Genetics and Development, Columbia University, New York, NY 10032, USA; 2 Department of Biochemistry and Molecular Biophysics, Columbia University, New York, NY 10032, USA

## Abstract

**Background:**

Neuronal topographic map formation requires appropriate selection of axonal trajectories at intermediate choice points prior to target innervation. Axons of neurons in the spinal cord lateral motor column (LMC), as defined by a transcription factor code, are thought to innervate limb target tissues exclusively. Axons of the medial and lateral LMC divisions appear to execute a binary decision at the base of the limb as they choose between ventral and dorsal limb trajectories. The cellular logic that guides motor axon trajectory choices into non-limb tissues such as the ventral flank remains unclear.

**Results:**

We determined the spinal cord motor column origin of motor nerves that innervate ventral flank tissues at hindlimb level. We found unexpectedly that a subset of medial LMC axons innervates ventral non-limb mesenchyme at hindlimb level, rather than entering ventral limb mesenchyme. We also found that in a conditional *BmprIa *mutant where all ventral hindlimb mesenchyme is converted to a dorsal identity, all medial LMC axons are redirected into the ventral flank, while lateral LMC axons innervate the bidorsal limb.

**Conclusion:**

We have found that medial LMC neurons innervate both ventral flank and limb targets. While normally only a subset of medial LMC axons innervate the flank, all are capable of doing so. Furthermore, LMC axons execute a ternary, rather than binary, choice at the base of the limb between ventral flank, ventral limb and dorsal limb trajectories. When making this choice, medial and lateral LMC axons exhibit different and asymmetric relative preferences for these three trajectories. These data redefine the LMC as a motor column that innervates both limb and body tissues.

## Background

The precision of neural circuits requires stereotypic patterns of neuronal connectivity, which are often organized as topographic maps. Studies of how the point-to-point connections between spinal cord motor neurons and their targets are generated have revealed much about how such maps are constructed [1-3]. One principle to emerge from these studies is that functionally related neurons extend axons along shared trajectories that are precisely and accurately subdivided at intermediate choice points [4,5]. Deciphering the logic underlying these trajectory choices is thus critical to understanding how these connectivity patterns are established.

The overall map between motor neurons and their peripheral targets is well described [3,6-8]. The motor neurons are organized mediolaterally into medial and lateral motor columns (MMC and LMC) and their divisions. Medial MMC neurons innervate dorsal axial muscles. At thoracic levels, lateral MMC neurons innervate ventral body wall muscles, and autonomic motor neurons of the intermediolateral column (IML, column of Terni in birds) innervate sympathetic postganglionic neurons. At limb levels, lateral LMC neurons innervate dorsal limb muscles while medial LMC neurons innervate ventral limb muscles. The motor columns are further subdivided into pools, and each motor pool innervates an individual muscle [3]. These descriptions are generally accurate, although possibly incomplete, as the identity of motor neurons that innervate many muscles, particularly outside of the limb, has not been examined in detail.

Current models suggest that shared intrinsic properties of neurons in a columnar division guide common trajectory decisions required of all axons projecting from that division [3]. With the exception of some cervical motor populations, all motor axons exit the ventral root of the spinal cord along a ventrolateral trajectory. Divisional populations deviate from this shared path at progressively more distal choice points. At limb axial levels the final divisional decision is made in the motor plexus at the base of the limb, where LMC axons assume trajectories into either dorsal or ventral limb mesenchyme.

The discovery that combinations of LIM homeodomain transcription factors identify motor neurons within columnar divisions led to the idea that the columnar identity of a motor neuron as defined by this transcription factor code is predictive of its axonal trajectory [3,9]. Consistent with this, neurons whose cell bodies are misplaced within morphological columns relative to their axonal trajectories nonetheless express columnar transcription factors appropriate to their trajectories [9]. Gain and loss of function genetic experiments demonstrate that these transcription factors impose specific pathfinding behaviors on motor axons [10,11], and do so largely by regulating expression of axon guidance receptors [12,13].

Experiments designed to test how trajectories into limb mesenchyme are chosen by axons of LMC neurons have invariably found both medial and lateral LMC axons entering the limb. This is the case for limbs that are surgically truncated, rotated or duplicated along the dorsal-ventral axis [4,14-17]; for mutants in which limb mesenchymal dorsoventral identity or guidance cue expression is altered [11,18-20]; for mutants in which LMC axonal guidance receptor expression is altered [12,19-22]; and for embryos in which spontaneous neural activity is inhibited [23]. These results, in combination with descriptions of the neuromuscular topographic map [6-8], have led to the idea that at the base of the limb, LMC axons have only two options: they make a binary choice between trajectories into either dorsal or ventral limb mesenchyme [3,24-26].

The contributions of both intrinsic properties of LMC motor neurons and extrinsic guidance cues to this trajectory choice have been further defined. Surgical experiments demonstrate that the choice is active, and is regulated locally by signals generated at the choice point [4,14-16]. Molecular genetic experiments have identified primarily [12,18,19,27,28], but not exclusively [20], repulsive ligand:receptor combinations that influence this choice. The emerging model is that the LIM homeodomain code imparts selective insensitivity to peripheral guidance cues until axons arrive at the base of the limb [3,13]. Differential sensitivity to guidance cues reflecting limb dorsoventral pattern then guides medial and lateral LMC axons as they choose between dorsal and ventral limb trajectories [11,12,18-20,28].

While appealing, it is not clear if this model can account for the selection of trajectories by all motor axons that reach the base of the limb. Several nerves (iliohypogastric, ilioinguinal and genitofemoral) that project from the most rostral lumbar segments (L1–L2) of the spinal cord innervate lateral abdominal body wall (external oblique, internal oblique and transverse) and genital (cremaster) muscles [29,30]. The motor axons that ultimately form these nerves extend through the plexus into ventral flank mesenchyme. These trajectories are not typical of medial MMC, IML, medial LMC or lateral LMC axons, which are all present at this axial level.

We investigated the columnar identity of the ventral flank nerves and found that they originate within the medial LMC. This result indicates there is a third trajectory, in addition to the limb trajectories, available to LMC axons, one that binary choice models do not accommodate. To investigate how peripheral cues influence LMC axon trajectory decisions, we removed the ventral limb mesenchyme using a conditional *bone morphogenetic protein receptor Ia *genetic model (*BmprIa*^*flox*/-^) [31], in which all hindlimb mesenchyme has a dorsal identity. When medial LMC axons are confronted with this bidorsal limb mesenchyme, they do not enter the limb, and are redirected into the ventral flank. In contrast, lateral LMC axons enter the bidorsal hindlimb and populate both dorsal and ventral nerve branches. Our results show that motor axons have a ternary, not binary, choice of trajectories at the base of the hindlimb, and reveal that medial and lateral LMC axons have different and asymmetric preferences for trajectories that enter ventral flank, ventral limb or dorsal limb mesenchyme. Our findings redefine LMC columnar identity as predictive of trajectories that include both body and limb tissues.

## Results

### Medial LMC axons project to the ventral flank mesenchyme

**Figure 1:**
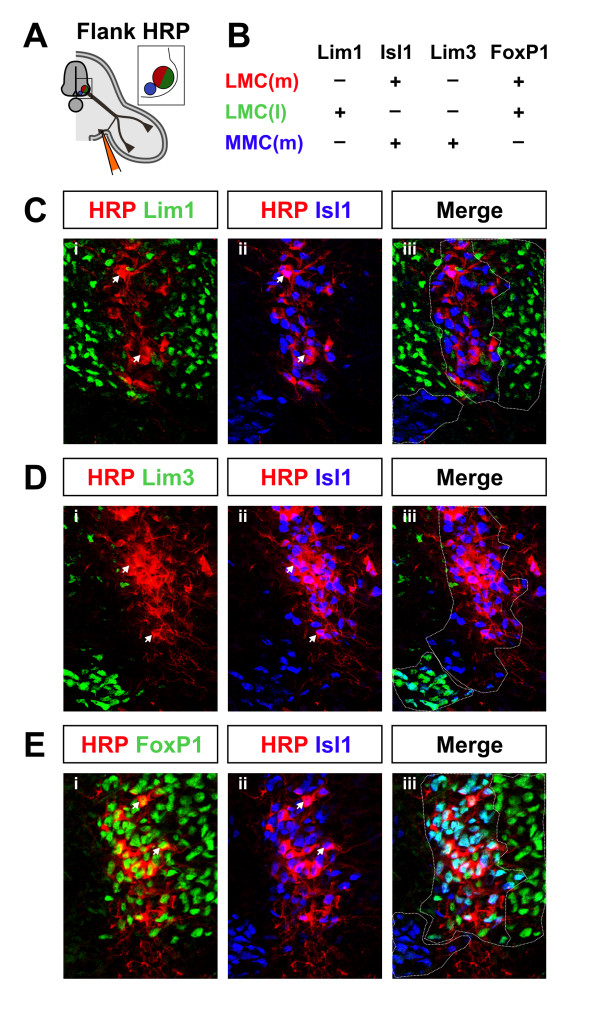
Medial LMC axons innervate the ventral flank mesenchyme. The columnar origin of neurons innervating the ventral flank mesenchyme was defined by HRP or RDA retrograde labeling at E13.5. **(a) **Schematic cross section at hindlimb level, showing limb and flank axonal trajectories and approximate location of the retrograde label injection. Inset: relative positions of motor columns in C-E. **(b) **Transcription factor combinations expressed by medial LMC (LMC(m)), lateral LMC (LMC(l)) and medial MMC (MMC(m)) neurons. **(c,d,e) **Representative images showing HRP colocalization with Isl1, and not Lim1 (c) or Lim3 (d), but with FoxP1 (e), in triply immunostained sections. Note that images show representative staining patterns and marker colocalization, and are not intended to be quantitative. Arrowheads: HRP+ neurons colabeled with nuclear markers. Dotted lines (c-e) delineate columnar division borders. n = 5 embryos.

To determine the columnar identity of the motor neurons whose axons project into the ventral flank at hindlimb axial levels, we retrogradely labeled these neurons by horseradish peroxidase (HRP) or lysinated tetramethylrhodamine dextran (RDA) injection [24] into ventral flank mesenchyme of E13.5 mouse embryos (Figure 1). HRP+ and RDA+ cell bodies are restricted to rostral lumbar regions, consistent with the described trajectories of the relevant nerves. Retrogradely labeled neurons express predominantly the medial LMC marker Isl1, but not the medial MMC marker Lim3 or the lateral LMC marker Lim1 (Figure 1c,d) [9]. They also express FoxP1, which marks LMC but not MMC motor neurons (Figure 1e; Jeremy Dasen and Thomas Jessell, Columbia University, personal communication) [32]. The ventral flank nerves are thus composed of motor axons that originate from medial LMC neurons.

To determine whether these neurons exist within a single motor pool, we asked whether the retrogradely labeled neurons express additional transcription factor markers that label subsets of medial LMC neurons [33,34]. We could readily detect labeled Nkx6.1+ and labeled Nkx6.1- neurons, and could occasionally detect labeled Er81+ neurons (Additional file 1). Together these data indicate that neurons that project along ventral flank trajectories likely derive from multiple motor pools, which is consistent with the observation that the nerves innervate multiple target muscles.

A subset of medial LMC neurons, as defined by their LIM homeodomain transcription factor code, thus unexpectedly project not to the limb, but to the ventral flank, after leaving the anterior plexus. This raises the possibility that this non-limb trajectory might be available to other medial LMC or perhaps all LMC axons. If so, then additional medial LMC neurons might also project to the ventral flank mesenchyme in appropriate mutant contexts.

### *BmprIa*^*flox*/- ^mutant embryos have completely bidorsal hindlimbs

We reasoned that a mutant lacking ventral limb mesenchyme, the predominant medial LMC axonal target, is one scenario in which additional ventral flank projections might be observed, as medial LMC axons would, of necessity, innervate an alternative mesenchymal territory. *Brn4-cre*^*Tg*/-^, *BmprIa*^*flox*/- ^(*BmprIa*^*flox*/-^) mice carry a conditional null allele of the *BmprIa *gene that is inactivated to homozygosity by Cre recombinase expressed under the control of a transgenic *Brn4 *promoter [31]. In these mice the ventral hindlimb mesenchyme is transformed to an apparently dorsal identity, while forelimb dorsoventral polarity is normal [31,35,36]. While *BmprIa*^*flox*/- ^mutant hindlimbs are small, they nonetheless include proximal and intermediate limb tissue [31]. This mutant is thus a promising candidate in which to look for additional LMC axons innervating the ventral flank.

The dorsoventral projection choice is controlled by local signals [15,16,37]. Thus, we first confirmed that at the time of the choice in the *BmprIa*^*flox*/- ^mutant no residual ventral limb mesenchyme is present near the choice point, as previous studies did not establish this [31,36]. We defined a set of molecular markers for evaluating the dorsoventral identity of the proximal limb mesenchyme. Our criteria were: (1) that each marker is expressed either throughout the dorsoventral extent of the limb or is restricted to dorsal or ventral limb; and (2) that the markers share a proximal boundary adjacent to the plexus as axons begin entering the limb mesenchyme  (E10.5–11.0 in hindlimb, E10.0–10.5 in forelimb) [11,16]. The markers in combination (the 'dorsoventral code') should define the limb mesenchyme as dorsal or ventral with respect to the projection choice and account for all topography along the limb dorsoventral axis.

**Figure 2:**
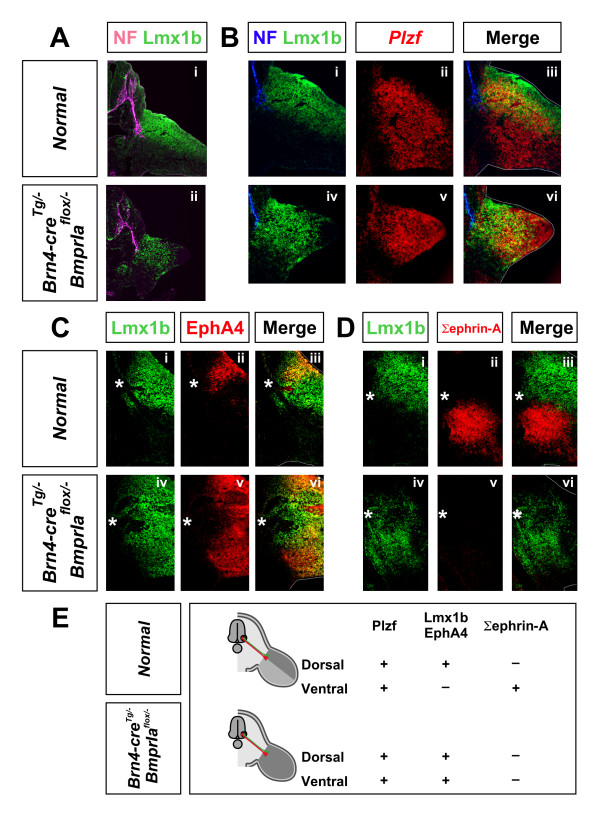
*BmprIa*^*flox*/- ^hindlimbs are bidorsal at and around the time of the motor axon dorsoventral projection choice. The dorsoventral character of limb mesenchyme was defined using molecular markers. *Brn4-cre*^*Tg*/-^, *BmprIa*^*flox*/- ^(*BmprIa*^*flox*/-^) mutant hindlimbs are completely dorsalized. **(a-d) **Upper panels: control hindlimbs. At E11.0 axons have reached the base of the hindlimb (a,b). *Plzf *is a general limb marker (b), and Lmx1b is a dorsal limb marker (a,b). EphA4 marks proximal dorsal mesenchyme (c) and Σephrin-A marks ventral mesenchyme (d) at E11.5. Note coincidence of proximal expression borders. Lower panels: *BmprIa*^*flox*/- ^mutant hindlimbs. Marker expression demonstrates they are completely dorsalized (Lmx1b+ Σephrin-A-) (a,b,d) and that they are proximally dorsal (EphA4+) (c). **(e) **Summary of molecular dorsoventral code marker expression in control (upper) or bidorsal *BmprIa*^*flox*/- ^mutant (lower) hindlimb. RNA *in situ *hybridization (*Plzf*), immunostaining (Lmx1b, EphA4) and AP fusion protein staining (Σephrin-A) were performed on adjacent transverse cryosections and images were digitally superimposed. n = 12 (a,b), 8 (c), 5 (d) embryos. Top, dorsal; left, medial. Dotted lines: limb outline. NF, neurofilament. Asterisks indicate the position of the plexus.

We screened candidate gene expression patterns, and identified a collection of markers that fit these criteria (Figure 2; Additional file 2). The transcription factor *Plzf *[38], marks the entire limb mesenchyme (Figure 2b). The transcription factor *Lmx1b *[39,40] marks dorsal limb mesenchyme (Figure 2a,c), and the *EphA4 *receptor marks proximal dorsal limb mesenchyme (Figure 2c) [21]. *ephrin-A2*, *ephrin-A3 *and *ephrin-A5*, which encode ligands for Eph receptors, are expressed in the ventral limb [12,22,28,41], and the sum (Σ) of ephrin-A proteins, detected using an EphA4-Fc soluble fusion protein [42], marks ventral limb (Figure 2d). *Plzf*, Lmx1b, *EphA4 *and Σephrin-A thus together define limb mesenchyme identity with respect to the dorsoventral axonal projection choice: dorsal limb mesenchyme is *Plzf*+ Lmx1b+ EphA4+ Σephrin-A- and ventral limb mesenchyme is *Plzf*+ Lmx1b- EphA4- Σephrin-A+ (Figure 2e). In normal limbs each territory occupies approximately half of the mesenchyme.

We used these markers to examine the dorsoventral character of *BmprIa*^*flox*/- ^mutant limb mesenchyme. All of the *Plzf*+ hindlimb mesenchyme is Lmx1b+ (Figure 2b) and is also Σephrin-A- (Figure 2d). EphA4 is present throughout the dorsoventral extent of the hindlimb mesenchyme and, notably, its proximal ventral expression boundary coincides with that of Lmx1b (Figure 2c). Taken together, these results provide evidence that all of the mutant hindlimb mesenchyme is dorsal (*Plzf*+ Lmx1b+ Σephrin-A-) and that at the base of the limb it is proximally dorsal (EphA4+). By contrast, *BmprIa*^*flox*/- ^mutant forelimb mesenchyme has normal dorsoventral polarity, consistent with previous reports (Additional file 2) [31]. Thus, relative to the dorsoventral projection choice, the dorsal transformation of ventral mesenchyme in *BmprIa*^*flox*/- ^hindlimbs is complete.

### A dorsoventral axon branch point forms in *BmprIa*^*flox*/- ^mutant hindlimbs

**Figure 3:**
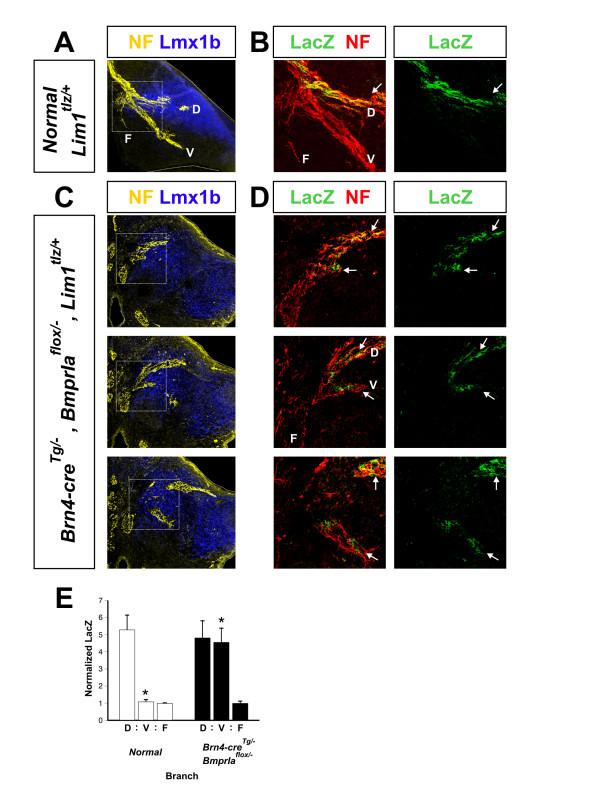
Both nerve branches contain lateral LMC axons in *BmprIa*^*flox*/- ^hindlimbs. Comparison of anterior plexus axonal projection patterns in control and mutant embryos. A *Lim1*^*tlz*^allele was used to label lateral LMC axons with LacZ. **(a,b) **E11.75 control hindlimb; overlays of two adjacent sections. (a) NF+ nerves form three branches at the base of the hindlimb, with only the dorsal branch entering Lmx1b+ dorsal limb mesenchyme. (b) Only the dorsal limb branch contains LacZ+ lateral LMC axons. **(c,d) **E11.75 mutant hindlimb; in consecutive coimmunostained sections, dorsal, ventral and flank branches can be followed. (c) NF+ nerves form three branches, two of which enter Lmx1b+ limb mesenchyme. (d) Both limb nerve branches contain LacZ+ lateral LMC axons, while the ventral flank branch does not. **(e) **LacZ immunoreactivity was quantified and normalized for neurofilament immunoreactivity in each nerve branch; low-level signal in ventral limb and flank branches likely represents weak non-specific staining. Values are presented in relative units that represent the normalized LacZ signal in each branch. The relative signals in normal limbs (white bars) were (mean ± SEM): 5.3 ± 0.9 (dorsal), 1.1 ± 0.2 (ventral), 1 ± 0.1 (flank); n = 5 embryos. The relative signals in mutant limbs (black bars) were: 4.8 ± 1 (dorsal), 4.6 ± 0.9 (ventral), 1 ± 0.2 (flank); n = 5 embryos. *P *= 0.00005 for ventral limb branches, two-tailed t test. *P < 0.00005. D, dorsal limb nerve branch; F, ventral flank nerve branch; V, ventral limb nerve branch. Arrows: LacZ+ nerves. Dotted lines: limb outlines. Boxed areas in (a,c) are enlarged in (b,d).

We next examined the early axonal trajectories into the bidorsal *BmprIa*^*flox*/- ^mutant limbs in mice also carrying the *Lim1*^*tlz *^allele, which directs *tau-lacZ *reporter expression only to axons of lateral LMC neurons [11]. At rostral axial levels of normal hindlimbs, neurofilament (NF) immunostaining, which labels all axons, marks a LacZ+ dorsal limb branch invading Lmx1b+ mesenchyme, a LacZ- ventral limb branch entering Lmx1b- mesenchyme and a LacZ- ventral flank branch (Figure 3a,b). Along the complete rostrocaudal extent of the *BmprIa*^*flox*/- ^hindlimbs, two LacZ+ branches invade Lmx1b+ limb mesenchyme, and a LacZ- branch projects ventrally, into the Lmx1b- flank (Figure 3c,d). Quantification of LacZ immunoreactivity in each branch supports these observations (Figure 3e). In *BmprIa*^*flox*/- ^forelimbs, there is no difference in the nerve trajectories compared to normal forelimbs (Additional file 3). Together, these data demonstrate that three branches form with normal timing in the bidorsal limbs, but that lateral LMC axons populate both limb branches equally, and are absent from the ventral flank branch. These data also provide evidence that lateral LMC axons perceive both halves of the *BmprIa*^*flox*/- ^mutant hindlimb as equivalently dorsal.

### Medial LMC axons are redirected from ventral limb to ventral flank

**Figure 4:**
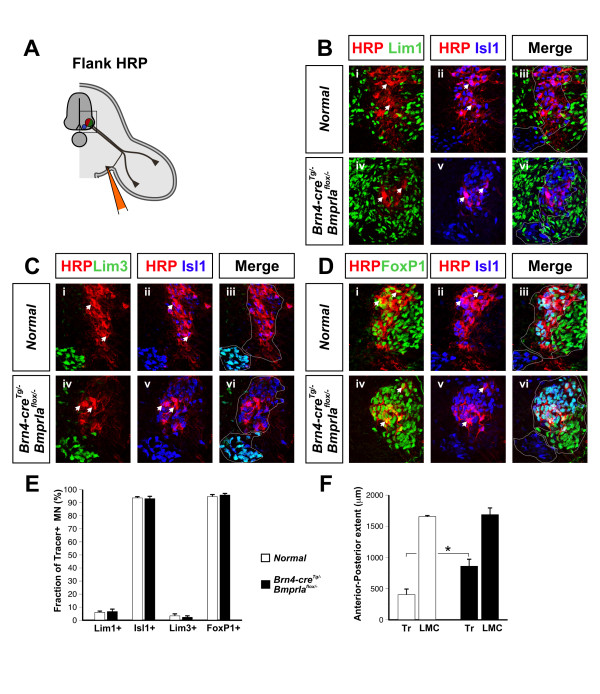
Medial LMC axons innervate the *BmprIa*^*flox*/- ^ventral flank mesenchyme. The columnar origin of neurons innervating the ventral flank mesenchyme at E13.5 was defined by HRP or RDA retrograde labeling. **(a) **Schematic of experiments shown in (b-d). **(b) **Flank nerves originate from medial LMC neurons in normal and mutant embryos, as Isl1+ medial LMC cells are readily HRP-labeled in triple coimmunostained sections. **(c) **Flank nerves in normal and mutant do not originate from medial MMC cells, as Lim3+ medial MMC cells are rarely HRP+ in triple coimmunostained sections. **(d) **Flank nerves in normal and mutant originate from medial LMC but not medial MMC as coimmunostained HRP+ Isl1+ FoxP1+ medial LMC cells were readily detected. In (b-d), upper panels are controls and lower panels are *BmprIa*^*flox*/- ^mutants. Arrowheads indicate representative HRP+ neurons colabeled with nuclear markers and dotted lines in (b-d) indicate columnar division outlines. **(e) **Quantification of labeling data shows that axons projecting to ventral flank mesenchyme in normal and *BmprIa*^*flox*/- ^mutant embryos are from Isl1+ Lim1- Lim3- FoxP1+ medial LMC neurons. The percentages of HRP+ or RDA+ cells were as follows. Isl1+ Lim1-: normal, 94 ± 1.2, n = 5 embryos, 648 neurons counted; mutant, 93 ± 2.1, n = 5, 388 neurons; *P *= 0.83. Lim3+: normal, 3.5 ± 1.8, n = 5, 515 neurons; mutant: 2.4 ± 1.4, n = 4, 283 neurons; *P *= 0.62. FoxP1: normal, 95 ± 1.5, n = 5, 479 neurons; mutant, 96 ± 1.5, n = 4, 262 neurons; *P *= 0.63). **(f) **Anterior-posterior length of the LMC is similar in normal and mutant E13.5 embryos, while the extent of the LMC labeled from the ventral flank is significantly increased in mutant embryos. LMC lengths (Isl1+/Hb9-GFP+ sections) were: normal, 1,658 ± 23 μm, n = 6 embryos; mutant, 1,690 ± 113 μm, n = 5; *P *= 0.745. Tracer extents (HRP+ or RDA+ sections) were: normal, 407 ± 96 μm, n = 5 embryos; mutant, 864 ± 116 μm, n = 5; *P *= 0.008.

To define directly the origin of the ventral flank motor branch in the *BmprIa*^*flox*/- ^mutant, we retrogradely labeled neurons projecting into the ventral flank. In both normal and *BmprIa*^*flox*/- ^mutant embryos, almost all HRP+ or RDA+ neurons express medial LMC markers (Figure 4a–d), while the remainder expresses lateral LMC or medial MMC markers. A similar 95% of labeled neurons also express the additional LMC marker FoxP1 (Figure 4d,e). This result is consistent with the *Lim1*^*tlz *^labeling experiments and provides evidence that in *BmprIa*^*flox*/- ^mutant embryos only medial LMC neurons innervate the ventral flank.

**Figure 5:**
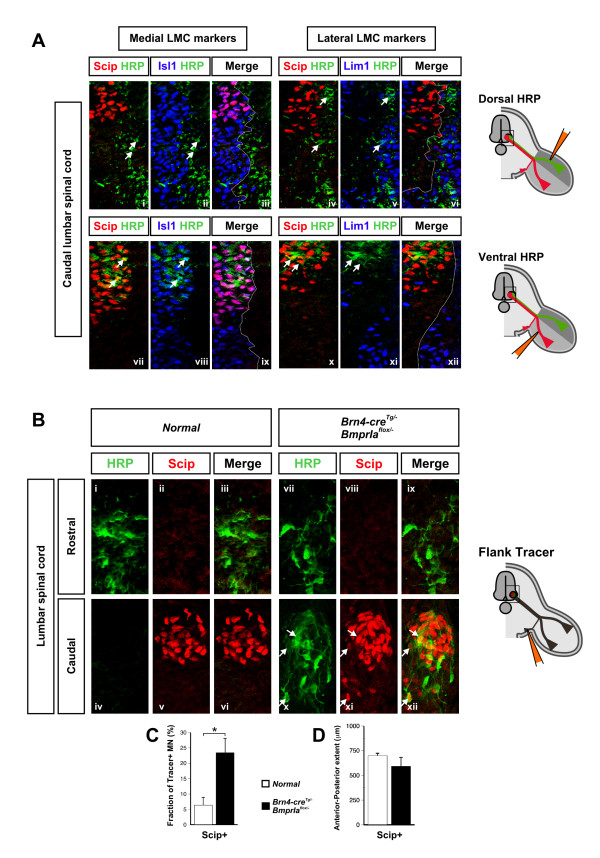
Medial LMC axons in the *BmprIa*^*flox*/-^ mutant are redirected from the ventral limb into the ventral flank. Scip immunostaining and retrograde labeling (HRP or RDA) of nerves at E13.5 was used to define the contribution of Scip+ neurons to each nerve branch. **(a)** Retrograde labeling from normal dorsal or ventral limb mesenchyme. Dorsal nerves originate from Scip- lateral LMC neurons (a, i-vi). HRP-labeled cells do not express Scip and are Isl1- Lim1+ lateral LMC cells (white arrows). Scip+ medial LMC cells contribute to ventral nerves (a, vii-xii). Many HRP-labeled cells express Scip and all Scip+ cells are Isl1+ Lim1- medial LMC neurons (white arrows). **(b)** Retrograde labeling from normal or mutant ventral flank mesenchyme. In normal and mutant embryos retrogradely labeled Scip- cells are readily detected in sections of rostral spinal cord (b, i-vi). Labeled cells are detected in caudal lumbar spinal cord only in *BmprIa*^*flox*/-^ mutants (b, vii-xii, white arrows). Many of these labeled neurons are also Scip+.  Similar staining patterns were observed in three embryos for each labeling experiment. Representative sections are shown of labeled motor columns co-immunostained for HRP and Scip. Experiments in (a,b) are diagrammed schematically to the right, and boxed areas show the regions of the images. **(c)** Quantification of flank retrograde labeling data. The fraction of HRP+ or RDA+ flank-labeled cells that are also Scip+ differs significantly (P < 0.02) between normal and mutant (normal: white bar, 6.4% ± 2.6%, n = 6 embryos, N = 1,018 labeled neurons; mutant: black bar, 23.6% ± 4.7%; n = 5 embryos, N = 784 labeled neurons). **(d)** The anteroposterior span of the Scip+ pool within the E13.5 lumbar LMC does not differ significantly between normal and mutant embryos (normal: 701 ± 26 μm, n = 6 embryos; mutant: 592 ± 94 μm, n = 5; P = 0.219).

To address whether these medial LMC axons innervating the mutant flank include axons that normally innervate the limb, we further analyzed the distribution and marker expression of the ventral flank-labeled neurons. While retrogradely labeled neurons in control embryos are restricted to more rostral lumbar regions and span about 25% of the rostrocaudal length of the LMC, in *BmprIa*^*flox*/- ^mutant embryos they are distributed more broadly along approximately 50% of the length of the lumbar LMC (Figure 5). Scip, a POU domain transcription factor [43,44], marks caudal lumbar medial LMC neurons that in control embryos are frequently colabeled with HRP following ventral limb injections (Figure 5) but infrequently (about 6% of labeled cells) following ventral flank injections (Figure 5). However, about 24% of labeled motor neurons are also Scip+ following ventral flank injections in *BmprIa*^*flox*/- ^mutants (Figure 5). The Scip+ population is equivalent in rostrocaudal extent and location in control and mutant embryos, as is the extent of the LMC itself, indicating that the differences are not due to an expansion of the Scip+ population, or gross changes in LMC organization (Figure 5). Scip thus marks caudal lumbar medial LMC neurons that normally project to ventral limb mesenchyme but whose axons enter the ventral flank in *BmprIa*^*flox*/- ^mutant embryos. These results provide direct evidence that medial LMC axons are redirected from the ventral limb to the ventral flank when confronted with only dorsal limb mesenchyme.

While at hindlimb levels some medial LMC axons are rerouted to the ventral flank in *BmprIa*^*flox*/- ^mutants, other medial LMC axons might assume different trajectories, or perhaps stall at the base of the limb. NF immunostaining indicates that axons do not assume random trajectories in the peripheral non-limb mesenchyme. If axons stall, growth cones should accumulate in the hindlimb plexus. To address this possibility, we examined vesicular acetylcholine transporter (VAChT) expression [45], which preferentially labels distal regions of axons, shortly after the dorsoventral projection choice. We did not detect significant differences in VAChT staining between forelimbs or hindlimbs of normal and *BmprIa*^*flox*/- ^embryos (Additional file 4). These results provide evidence that axons extend through the hindlimb motor plexus of *BmprIa*^*flox*/- ^mutants.

**Figure 6:**
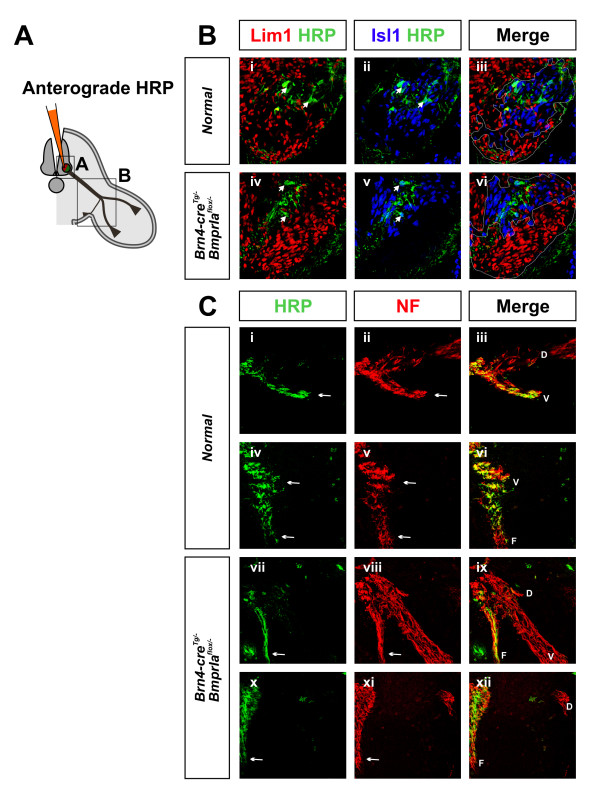
Medial LMC axons enter only the ventral flank nerve branch in the *BmprIa*^*flox*/- ^hindlimb. Anterograde labeling was used to determine the trajectories of axons originating within the medial LMC. **(a) **Schematic showing the dorsal approach used for the anterograde HRP labeling of spinal cord neurons at E12.5. Regions of interest in (b,c) are boxed. **(b) **Triple co-immunostaining showing spinal cords in which the vast majority of HRP+ cells are Isl1+ Lim1- medial LMC neurons. **(c) **In normal hindlimbs, both ventral flank and ventral limb branches are labeled with HRP (n = 5 embryos, label detected in ventral flank branch = 5/5, ventral limb branch = 5/5, dorsal limb branch = 0/5), while neurofilament labeling identifies all three nerve branches. In mutant hindlimbs, only the ventral flank branch is HRP+ (n = 5 embryos, ventral flank branch = 5/5, ventral limb branch = 0/5, dorsal limb branch = 0/5). Adjacent sections from anterior plexus are shown for both normal and mutant. Arrowheads: representative HRP+ Isl1+ neurons. Arrows: HRP+ nerve branches. Dotted lines: outlines of lateral and medial LMC. D, dorsal limb nerve branch; F, ventral flank nerve branch; V, ventral limb nerve branch.

To visualize the trajectories of medial LMC axons we anterogradely labeled medial LMC neurons by injecting HRP in the ventral medial spinal cord of E12.5 embryos. We only analyzed trajectories if an embryo had HRP+ medial LMC and not lateral LMC neurons (5 normal and 5 mutant embryos of 67 total embryos injected; Figure 6b). In normal embryos HRP labels primarily axons entering ventral limb and some entering ventral flank (Figure 6c). In contrast, in *BmprIa*^*flox*/- ^mutant embryos, only ventral flank axons are HRP+, while the limb branches are not labeled (Figure 6c). There also are no extra HRP+ axons at the branch point, further indicating that the medial LMC axons did not stall. These results provide evidence that medial LMC axons project only to the ventral flank of *BmprIa*^*flox*/-^mutant embryos, and do not perceive ventral flank and dorsal limb mesenchyme as equally permissive.

### Extra flank nerves are present in embryos with bidorsal limbs

**Figure 7:**
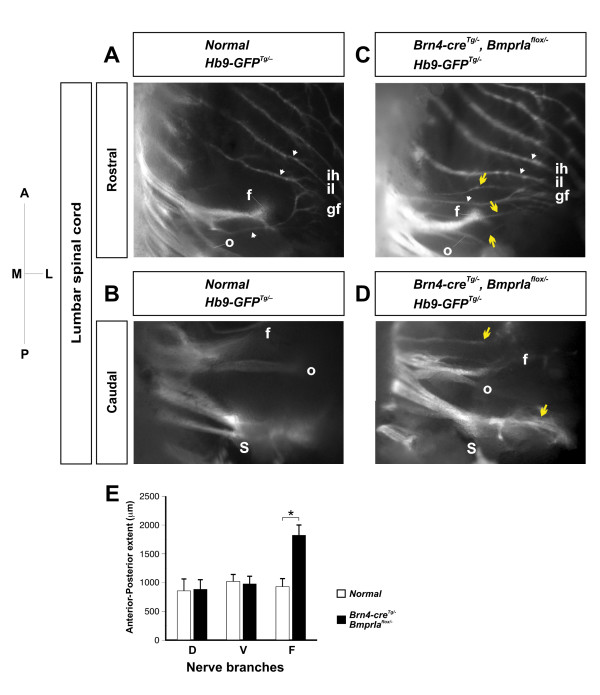
Lumbar spinal cord motor axon projections to the ventral flank. **(a-d) **E13.5 normal and *BmprIa*^*flox*/- ^embryos carrying an *Hb9-GFP *transgene imaged via indirect fluorescence. The embryos were eviscerated and the ventral abdominal wall was reflected to allow examination from a peritoneal aspect. Images of rostral (a,c) and caudal (b,d) regions are of different embryos, and in the caudal images the more rostral (for example, genitofemoral) nerves were removed during the dissection. In control embryos three major motor nerves (white arrows) project abdominally via the ventral flank from rostral segments of the lumbar spinal cord (a), while none project abdominally from more caudal segments (b). The limb nerves (f, femoral nerve; o, obturator nerve; S, sciatic plexus) turn along dorsal or ventral limb trajectories into the plane of the image. In mutant embryos, additional flank-projecting nerves (yellow arrows) are present at both rostral (c) and caudal (d) levels. **(e) **Quantification of nerve trajectories at E11.5–11.75. Transverse sections of normal and *BmprIa*^*flox*/- ^embryos carrying an *Hb9-GFP *transgene were scored for the presence of a GFP+ dorsal limb, ventral limb or ventral flank nerve branch. The combined continuous anteroposterior extent of sections containing nerves following each trajectory is presented. gf, genitofemoral nerve; ih, iliohypogastric nerve; il, ilioinguinal nerve. M, medial; L, lateral; A, anterior; P, posterior.

To compare directly the trajectories of the lumbar motor axons in normal and *BmprIa*^*flox*/- ^mutant embryos, we crossed an *Hb9-GFP *transgenic allele [46] into these mice. The *Hb9 *promoter drives green fluorescent protein (GFP) expression in motor neurons, but not in sensory neurons, allowing direct visualization of the motor axons. We scored the distribution of GFP+ axons projecting along ventral flank, ventral limb and dorsal limb trajectories in sections of E11.5–E11.75 embryos (Figure 7e). Axons entering either limb branch spanned similar extents of the embryonic anteroposterior axis in control and mutant embryos, consistent with the observation that the anteroposterior extent of the mutant limbs is not smaller at the time of axon arrival (data not shown). In contrast, axons assuming flank trajectories spanned twice as much territory in mutant embryos compared to controls. Perhaps not surprisingly, the anteroposterior extent of both limb and flank-projecting axons we detected by direct visualization was greater than that detected by retrograde labeling (Figure 5). This likely reflects the difficulty of quantitative retrograde labeling, and the conservative nature of the retrograde tracer injections necessary not to contaminate inappropriate nerve branches.

We also examined the trajectories of GFP+ axons that project along the lower abdominal wall in freshly dissected, intact E13.5 embryos (Figure 7). In control embryos (N = 8/8 embryos, 16/16 limbs) three nerves were detected projecting abdominally from the rostral lumbar spinal cord when viewed from a peritoneal aspect (Figure 7a). The two most rostral of these, the iliohypogastric and ilioinguinal nerves, project laterally from L1 and L2, and follow trajectories that parallel those of the more rostral subcostal and intercostal nerves. The third, the genitofemoral nerve, projects laterally upon leaving the rostral plexus in caudal L3 and curves anteriorly towards the ilioinguinal nerve. The ilioinguinal and genitofemoral trajectories together describe a roughly half-moon pattern with the ilioinguinal transcribing the meridian and the genitofemoral the perimeter. The genitofemoral leaves the plexus coincident with the femoral and obturator nerves that extend distally into the limb. More caudally no lumbar nerves follow flank trajectories after leaving the sciatic plexus, although many nerves extend into the limb (Figure 7b).

In *BmprIa*^*flox*/- ^mutant embryos (N = 3 embryos, 6 limbs) there are differences from controls both in the distribution and number of flank projections. The genitofemoral nerve projects more directly laterally, which results in more even spacing among the three major flank nerves (Figure 7c). In addition, one to three excess nerves project from rostral lumbar segments along abdominal trajectories, although with variable projection patterns that differ even within a single embryo. These fibers are located between the ilioinguinal and genitofemoral nerves or caudal to the genitofemoral nerve. A substantial population of novel GFP+ axons also extends abdominally from the sciatic plexus (Figure 7d). Nerves projecting along the femoral and obturator trajectories are visible, although reduced in size, and sciatic projections into the limb are also present. These data provide clear evidence that the ventral flank axons normally derive from rostral lumbar regions, and that when confronted with only dorsal limb mesenchyme, motor axons originating along the length of the lumbar spinal cord aberrantly choose ventral flank trajectories.

### Only lateral LMC axons enter the bidorsal *BmprIa*^*flox*/- ^hindlimb

**Figure 8:**
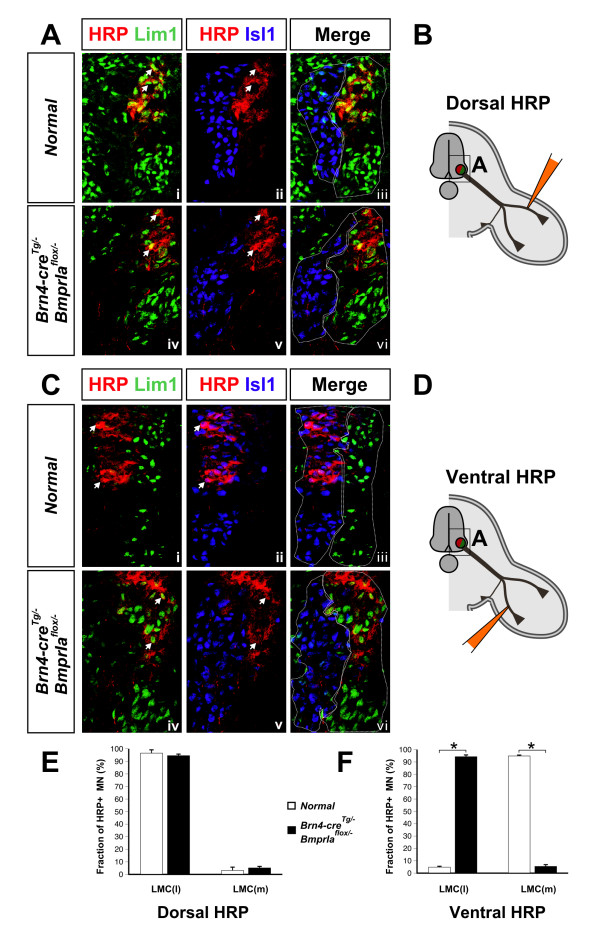
Only lateral LMC axons innervate *BmprIa*^*flox*/-^ hindlimb mesenchyme. HRP retrograde labeling at E13.5 identified spinal cord neurons that contribute to each limb nerve branch. **(a)** Dorsal limb nerve originates from the lateral LMC in normal and mutant as HRP+ Lim1+ lateral LMC cells are readily detected, while HRP+ Isl1+ cells are not, in triple co-immunostained sections. **(b)** Schematic of the experiment in (a). **(c)** Ventral limb nerve originates from the medial LMC in normal embryo as HRP+ cells are also Isl1+ Lim1-. In contrast, this nerve originates from lateral LMC neurons in the mutant as HRP+ cells are Lim1+ Isl1-. **(d)** Schematic of the experiment in (c). In (a-d) the upper panels show control embryos and the lower panels show *BmprIa*^*flox*/-^ mutants. Arrowheads indicate representative HRP+ Lim1+ or HRP+ Isl1+ neurons. Dotted lines in (a,c) indicate lateral and medial LMC outlines. Boxed areas in (b,d) indicate regions of interest in (a,c). **(e,f)** Quantification of (e) dorsal and (f) ventral retrograde labeling data. Lim1+ Isl1- HRP+ lateral LMC or Lim1- Isl1+ HRP+ medial LMC cells were counted in triply immunostained sections after retrograde labeling of control and *BmprIa*^*flox*/-^ hindlimbs, and are shown as percentages of total HRP+ cells. Dorsal limb HRP injection does not label significantly different populations in normal and mutant (normal: lateral LMC 97% ± 3%, n = 3 embryos, N > 150 HRP+ neurons; mutant: lateral LMC 95% ± 2%, n = 8 embryos, N > 350 HRP+ neurons; P = 0.6). Ventral limb HRP injection does label significantly different cell populations (normal: medial LMC 95% ± 1%, n = 6 embryos, N > 300 HRP+ neurons; mutant: medial LMC 6% ± 2%, n = 9 embryos, N > 400 HRP+ neurons; P < 0.00001). *P < 0.00001.

The anterograde and *Lim1*^*tlz *^labeling results together imply that lateral LMC neurons are the only motor neurons that innervate the bidorsal limb mesenchyme. To address this directly, we retrogradely labeled neurons innervating the *BmprIa*^*flox*/- ^mutant hindlimbs. HRP injections into dorsal hindlimb of either normal or *BmprIa*^*flox*/- ^mutant embryos at E13.5 label 95% to 97% lateral LMC neurons (Figure 8a,e). HRP injection into the ventral hindlimb of normal embryos labels 95% medial LMC neurons, as expected (Figure 8c,f). In contrast, ventral hindlimb injection of *BmprIa*^*flox*/- ^mutants labels lateral (94%), and not medial (6%), LMC neurons (Figure 8c,f). These data provide additional evidence that the motor components of both nerve branches that enter *BmprIa*^*flox*/- ^mutant hindlimb mesenchyme are composed solely of lateral LMC axons. Our anterograde labeling results (Figure 6) further support the idea that the failure to detect medial LMC axons by retrograde labeling from *BmprIa*^*flox*/- ^mutant hindlimbs is not due to a selective inability of the medial LMC axons to take up or transport the label, but rather is due to their absence from the limb mesenchyme.

### Altered LMC trajectories are due to changes in the *BmprIa*^*flox*/- ^hindlimb

Because the *Brn4-cre *transgene is expressed throughout the spinal cord, in addition to the limb ectoderm [31,35], we asked whether removing *BmprIa *from only the spinal cord might alter the LMC axonal trajectories. We first used molecular markers to label ventral progenitor or postmitotic motor neuron and interneuron populations at both brachial and lumbar levels of the spinal cord in *BmprIa*^*flox*/- ^mutants. We observed no significant difference between *BmprIa*^*flox*/- ^mutant and normal sibling progenitor or motor neuron populations at either axial level (Additional file 5). We next examined LMC motor projection patterns to *BmprIa*^*flox*/- ^forelimbs, which have normal dorsoventral patterning. *Lim1*^*tlz *^lateral LMC marking and retrograde labeling analyses from dorsal and ventral forelimb mesenchyme all show that the columnar origins of these axons are normal (Additional files 3 and 6). Thus removing *BmprIa *from the spinal cord does not alter the LMC dorsoventral trajectory choice at forelimb levels.

**Figure 9:**
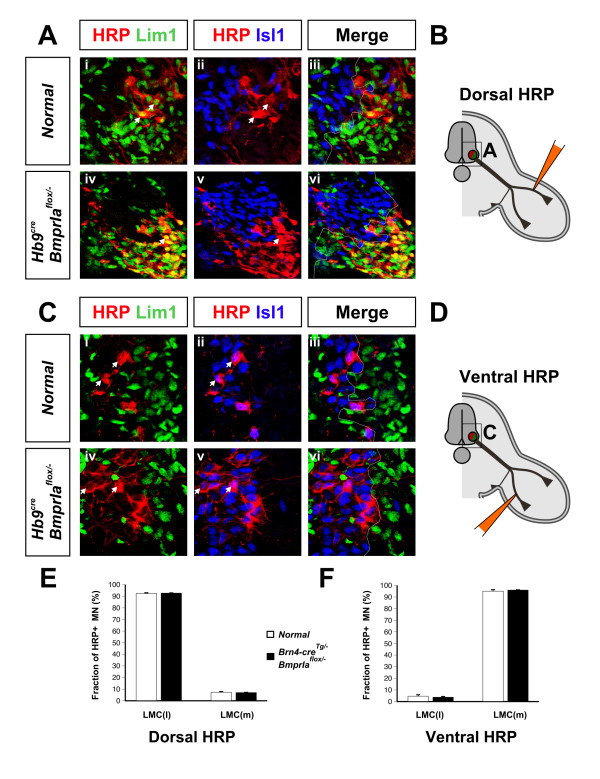
The motor innervation of *Hb9*^*cre*/+^, *BmprIa*^*flox*/- ^hindlimbs is normal. Retrograde labeling was used to determine the columnar origin of axons innervating *Hb9*^*cre*/+^, *BmprIa*^*flox*/- ^hindlimbs. **(a) **Dorsal nerves originate from Isl1- Lim1+ lateral LMC neurons in normal and *Hb9*^*cre*/+^, *BmprIa*^*flox*/- ^mutant embryos. **(b) **Schematic of the experiment in (a). **(c) **Ventral nerves originate from Isl1+ Lim1- medial LMC neurons in normal and *Hb9*^*cre*/+^, *BmprIa*^*flox*/- ^mutant embryos. **(d) **Schematic of the experiment in (c). **(e,f) **Quantification of dorsal (e) and ventral (f) retrograde labeling data. Lateral LMC cells were Lim1+ Isl1- HRP+; medial LMC cells were Lim1- Isl1+ HRP+. The percentage of labeled lateral or medial LMC cells marked by either dorsal or ventral limb HRP injection does not differ significantly between normal and mutant. Dorsal: lateral LMC 93% ± 0.9%, n = 8 embryos, N > 750 HRP+ neurons; mutant lateral LMC 93% ± 0.5%, n = 3 embryos, N > 450 HRP+ neurons; *P *= 0.79. Ventral: medial LMC 95% ± 1.5%, n = 5 embryos, N > 350 HRP+ neurons; mutant medial LMC 96% ± 0.9%, n = 3 embryos, N > 240 HRP+ neurons; *P *= 0.60.

To address directly whether removing *BmprIa *from lumbar LMC neurons alters their trajectories, we examined hindlimb LMC axonal projection patterns in *Hb9*^*cre*/+^, *BmprIa*^*flox*/- ^embryos. In these animals Cre is expressed in motor neurons and some ventral interneurons as they are becoming postmitotic, but is not expressed in the limbs (Additional file 7) [47]. It thus should remove *BmprIa *from most, if not all, LMC neurons prior to arrival of the axons at the base of the hindlimb. We found by retrograde labeling from dorsal or ventral hindlimb that there was no significant difference in the dorsoventral trajectory choice between *Hb9*^*cre*/+^, *BmprIa*^*flox*/- ^mutant and control embryos (Figure 9). Moreover, in *Hb9*^*cre*/+^, *BmprIa*^*flox*/- ^embryos the organization of the ventral spinal cord and dorsoventral pattern of the limb mesenchyme are normal (Additional file 7 and data not shown).

Multiple lines of evidence therefore indicate that inactivating *BmprIa *selectively in motor neurons does not perturb the limb motor axon projection patterns. These data include the normal projection patterns to *Brn4-cre*^*Tg*/-^, *BmprIa*^*flox*/- ^mutant forelimbs and to *Hb9*^*cre*/+^, *BmprIa*^*flox*/- ^mutant hindlimbs, as well as the normal generation of LMC neurons on both genetic backgrounds. These results provide strong support for the idea that the hindlimb projection defects observed in the *Brn4-cre*^*Tg*/-^, *BmprIa*^*flox*/- ^mutant are caused by altered guidance cues in the limb mesenchyme.

## Discussion

**Figure 10:**
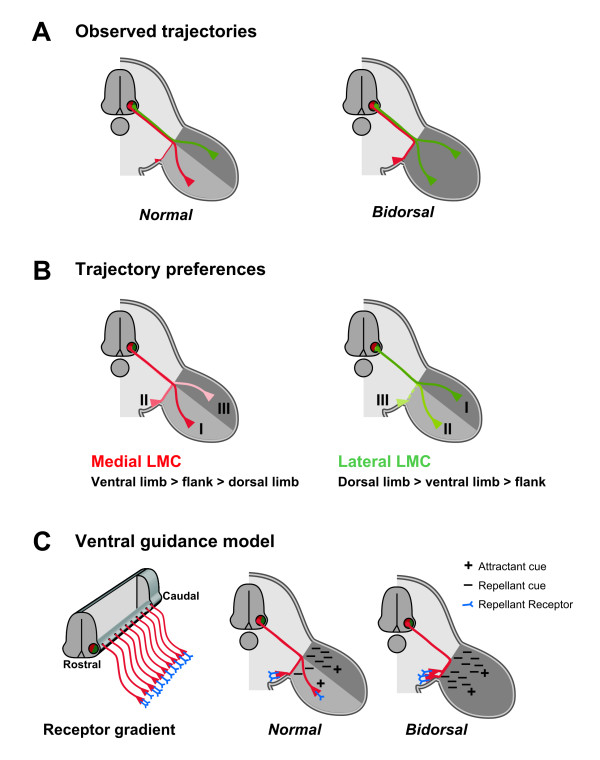
Summary of motor projections to bidorsal hindlimbs. **(a) **Observed trajectories of motor axons. In *Brn4-cre*^*Tg*/-^, *BmprIa*^*flox*/- ^mutant embryos with bidorsal hindlimbs, medial LMC axons do not invade the limb mesenchyme but are deflected to the ventral flank. Lateral LMC axons innervate both the dorsal and the ventral half of the bidorsal limb. **(b) **Trajectory preferences. LMC axons normally choose among three mesenchymal trajectories at the base of the limb, where they have different relative trajectory preferences (I > II > III for each branch; as it is unclear if lateral LMC axons ever enter the ventral flank, this branch is represented as a broken line). Medial LMC: ventral limb > ventral flank > dorsal limb. Lateral LMC: dorsal limb > ventral limb > ventral flank. **(c) **Model of guidance interactions. All medial LMC axons express receptor for a repellant guidance cue. A rostral subset of axons expresses high levels of receptor. In normal embryos, target mesenchyme expresses a step gradient of repellant cues: high levels in the dorsal limb, intermediate levels in the ventral limb, low levels in the ventral flank. Medial LMC axons expressing receptor at low levels are directed to the ventral limb by the high level of dorsal limb repellant, while the medial LMC axons expressing the receptor at high levels are directed to the flank by the intermediate levels of ventral limb repellant. All medial LMC axons also express receptor for an attractant cue expressed in limb mesenchyme. In bidorsal embryos, both limb halves express the repellant cue at high levels. Consequently, both subpopulations of medial LMC axons are directed to the flank. Blue (Y): receptor for repellant cue. (-): repellant guidance cue. (+): attractant guidance cue. Attractant receptor expressed on all medial LMC axons is not shown. Red axons: medial LMC. Green axons: lateral LMC.

We investigated the relationship between the columnar identity of motor neurons, as defined by a transcription factor code, and the projection decisions made by their axons as they reach the base of the limb. We found that the ventral flank mesenchyme is innervated by neurons of the medial division of the lateral motor column, which were previously thought to project only into the limb mesenchyme. We also found that while axons of lateral LMC neurons enter the *BmprIa*^*flox*/- ^bidorsal limb mesenchyme and assume both dorsal and ventral trajectories, those of medial LMC neurons do not enter the limb mesenchyme, and are directed into the ventral flank mesenchyme (Figure 10a). These results support the idea that at hindlimb levels LMC axons project to both flank and limb tissues, and while doing so they normally choose between three, not two, mesenchymal trajectories: into ventral flank, into ventral limb, or into dorsal limb. Our data also provide evidence that the relative preference for each trajectory differs significantly between medial LMC axons and lateral LMC axons. We discuss these ideas in the context of previous studies investigating the logic that underlies these projection decisions.

### Ventral flank mesenchyme is innervated by medial LMC neurons

We used multiple approaches to identify the motor neurons that extend axons into the ventral flank mesenchyme at hindlimb levels. All of them reveal that medial LMC neurons normally innervate both ventral limb and ventral flank mesenchyme. We therefore propose a change in the predictive value of the LIM homeodomain code regarding LMC motor axons [3,9]: Lim1- Isl1+ Lim3- medial LMC neurons should project to both ventral flank and ventral limb.

The axons that enter the hindlimb ventral flank are likely ultimately to form the motor components of the iliohypogastric, ilioinguinal and genitofemoral nerves [29,30]. In mammals these nerves arise from segments T12–L2 and they are the three most rostral nerves that project ventrally from the rostral hindlimb motor plexus. The motor components of the iliohypogastric and ilioinguinal nerves innervate lower abdominal muscles, while the sexually dimorphic genitofemoral nerve innervates the male cremaster muscle, which controls the position of the testes within the scrotum [48-50]. Previous experiments have not directly addressed the columnar origin of these nerves. However, retrograde labeling of the genitofemoral nerve from the cremaster muscle in rats marked a longitudinal distribution of somas in rostral lumbar regions [49], while ventral abdominal muscle labeling experiments consistently mark rostral lumbar motor pools [51-53]. Electrophysiological studies in humans demonstrate upper motor control of the genitofemoral nerve, and some degree of voluntary control [54]. These results are consistent with our identification of the ventral flank-projecting axons as somatic motor axons projecting from the rostral portion of the medial LMC.

### Relationship of medial LMC and lateral MMC neurons

Intriguingly, our observations support the idea that the trajectory preferences of lumbar medial LMC axons and thoracic lateral MMC axons are similarly regulated [9]. These neurons have the same LIM homeodomain transcription factor code and similar mediolateral somatic settling positions, and the ventral flank trajectory we documented for some medial LMC axons parallels that of lateral MMC axons. These axons make a series of similar projection decisions, ignoring both the dorsal trajectory of the medial MMC and the ventromedial trajectory of the autonomic motor neurons, before assuming a distal trajectory into ventral lateral plate mesodermal derivatives [9,13]. Our results also suggest that lateral MMC axons might perceive ventral flank and limb mesenchyme as similarly permissive, which might explain why they readily innervate ectopic thoracic limbs [55] or normal limbs when the axons are caudally displaced [2,4]. However, these populations are not a simple continuum, as LMC, but not lateral MMC, neurons express FoxP1 and Raldh2, for example [32,56]. It will be interesting to learn more about what these populations have in common, and how they diverged.

### LMC axons make a three-way projection decision at the base of the limb

Our data reveal that a trajectory into ventral flank mesenchyme is an option for LMC axons, which raises the question of why its significance was not previously appreciated. One likely reason is that relatively few motor axons normally enter the ventral flank mesenchyme, and thus they have not been studied as extensively as the axons that innervate the limb. Furthermore, previous experiments investigating initial limb innervation decisions always found that LMC axons entered the limb mesenchyme [2,11,12,15,16,19-23]. In addition, when motor innervation patterns were examined in apparently bidorsal chick hindlimbs generated surgically, they were found to be normal [17], in contrast to the situation in the bidorsal *BmprIa*^*flox*/- ^mutant hindlimbs. This might reflect a bona fide difference in the behavior of chick and mouse motor axons, although more likely small amounts of ventral limb mesenchyme remained near the dorsoventral choice point following the surgical manipulations, leading to a normal projection choice [37]. Together, these data led to the idea that at the base of the limb LMC axons make a binary choice between either dorsal or ventral limb trajectories [24,25]. Nonetheless, our data reveal that the motor axons execute a ternary, not a binary, choice of trajectories at hindlimb axial levels.

Whether lateral LMC axons enter ventral flank mesenchyme in existing mutants is not clear. We did not find lateral LMC axons making this choice when the hindlimbs are bidorsal. Similarly, no lateral LMC axons were described entering the ventral flank in *Lmx1b*^-/- ^mice, which have biventral hindlimbs [11], or in *EphA4*^-/- ^embryos, in which apparently all LMC axons are directed to ventral limb mesenchyme [21]. However, lateral LMC axons assuming this trajectory might have gone unnoticed, as the ventral flank nerve branch was not directly examined in the *Lmx1b*^-/- ^and *EphA4*^-/- ^mutants. One interesting question is why LacZ+ lateral LMC axons are not substantially redirected into the ventral flank mesenchyme of *Lim1*^*tlz*/+^, *Lmx1b*^-/- ^embryos, as they are repulsed by ephrin-A ligands that are uniformly high across the hindlimb of this mutant [11,12]. Perhaps attractive cues in limb mesenchyme such as glial-derived neurotrophic factor (GDNF) [20] can overcome these repellant signals, or an additional repulsive cue in the ventral flank makes this territory even less permissive than ventral limb mesenchyme to lateral LMC axons. Regardless, it appears that axon entry into limb mesenchyme is not a default trajectory for LMC axons, but rather involves an active choice.

The comparative behavior of medial and lateral LMC axons is informative about the regulatory logic guiding their trajectory choices. One might anticipate that peripheral guidance cues influence medial and lateral LMC axons in a similar but opposite fashion. If this were so, since lateral LMC axons populate dorsal and ventral nerve branches in equal proportion in *Lmx1b*^-/- ^mutants with biventral hindlimbs [11], then medial LMC axons should project equally to dorsal and ventral limb in *BmprIa*^*flox*/- ^mutants with bidorsal hindlimbs. However, medial LMC axons instead project to the ventral flank, indicating that medial and lateral LMC axonal trajectories are guided asymmetrically.

We can order the relative preference of the three trajectories from the perspective of axons in either LMC division (Figure 10b). From the perspective of medial LMC axons, the normal preference for most is to innervate ventral limb mesenchyme, with the remainder innervating the ventral flank. In the absence of ventral limb mesenchyme, ventral flank mesenchyme is strongly preferred over dorsal limb. From the perspective of lateral LMC axons, the normal preference is to innervate dorsal limb mesenchyme, and in its absence, to innervate ventral limb mesenchyme. The least favored trajectory appears to be into the ventral flank. Thus, the relative trajectory preferences for LMC axons are neither identical nor mirror images, and appear for medial LMC as: ventral limb > ventral flank > dorsal limb, and for lateral LMC as: dorsal limb > ventral limb > ventral flank.

The trajectory assumed by LMC axons might follow from one ternary choice between three options, or from sequential binary decisions. All three hindlimb LMC trajectories diverge at roughly the same proximodistal position within the hindlimb plexus (Figure 2). This contrasts with the trajectory assumed by some medial LMC axons that extend from the brachial plexus into forelimb level ventral flank mesenchyme, and that ultimately innervate the *latissimus dorsi *and *cutaneus maximus *muscles [57,58]. The forelimb ventral flank branch clearly diverges from the ventral limb branch after the axons of both branches have entered the ventral limb mesenchyme (for example, Additional file 3). This morphology implies that the decision to assume the forelimb flank trajectory is taken after the decision to enter the ventral limb mesenchyme. In contrast, the morphology of the hindlimb plexus favors a single ternary choice.

### A model for the three-way choice

The existence of both attractive and repulsive cues that guide lateral LMC axons provides a framework for thinking about how medial LMC axons are directed to ventral limb, and how a subset of them is guided to the ventral flank. In one model (Figure 10c), attractive guidance cues are present throughout the limb mesenchyme, but not in the ventral flank, and a step gradient of a repulsive cue is also present. The dorsal limb mesenchyme expresses the repellant at the highest level, the ventral limb mesenchyme at an intermediate level and the ventral flank mesenchyme at the lowest level. All medial LMC neurons express receptors for these cues, with a subset expressing the repellant receptor at higher levels. Upon arrival at the base of the limb, the growth cones integrate the attractive and repulsive cues and assume the most permissive trajectory. So long as the repellant activity in the dorsal limb mesenchyme dominates, all medial LMC axons are directed ventrally. In contrast, the moderate repellant level in ventral limb mesenchyme is permissive to most medial LMC axons, although the subset of axons with the highest level of the repellant receptor is directed more ventrally, into the flank mesenchyme. This model predicts that the limb dorsoventral patterning system controls repulsive cue levels, and thus in a completely dorsalized limb, such as in the *BmprIa *mutant, the entire limb mesenchyme expresses high levels of repulsive cue, and directs all medial LMC axons to ventral flank tissue.

If a subset of medial LMC neurons expresses more repulsive receptor, this leads to the question of how this subset is specified. The neurons that normally contribute to the ventral flank nerves are located in rostral segments of the lumbar spinal cord. Higher receptor expression levels therefore might be established by the system that patterns the anterior-posterior axis of the spinal cord [59], perhaps influenced by differential expression of pool-specific Hox genes [44] that act prior to overt segregation of the cell bodies into motor pools. Interestingly, the distribution of peripheral guidance cues might be established via another axial patterning system, since the dorsal limb, ventral limb and ventral flank mesenchyme are mediolaterally-arrayed derivatives of the lateral plate mesoderm [60]. While this model can account for the observed behavior of the medial LMC axons, others are equally plausible. Whatever the mechanism, it must make all medial LMC axons competent to assume ventral flank trajectories, while compelling only a few to do so.

## Conclusion

We found that neurons of the LMC, long thought to innervate only limb tissues, actually innervate tissues beyond the limb, such as the ventral flank. To select appropriate trajectories, LMC axons execute a three-way choice between ventral flank, ventral limb and dorsal limb targets. These findings provide new insights into the cellular logic used in guiding trajectory decisions, uncover a novel function for the LMC and illuminate the relationship between LMC axons and those of other motor columns.

## Methods

### Mouse mutants

Mice carried the following alleles in various combinations: *BmprIa*^*KO *^[61], *Brn4-cre*^*Tg *^[35], *BmprIa*^*flox *^[62], *Hb9*^*cre *^[47], *Hb9-GFP*^*Tg *^[46] and *Lim1*^*tlz *^[11]. Mice were maintained on mixed C57B6.J and 129SvEv backgrounds. *Brn4-cre*^*Tg*/-^, *BmprIa*^*flox*/- ^and *Hb9*^*cre*^, *BmprIa*^*flox*/- ^embryos were generated by intercrossing *Brn4-cre*^*Tg*/-^, *BmprIa*^*KO*/+ ^or *Hb9*^*cre*^, *BmprIa*^*KO*/+ ^and *BmprIa*^*flox*/*flox *^parents. *Brn4-cre*^*Tg*/-^, *BmprIa*^*flox*/- ^embryos were identified by hindlimb morphology [31]. All other allelic combinations from *Brn4-cre *matings were indistinguishable from wild-type controls and were designated normal [31].

Noon of the day a mating plug was observed was designated embryonic day 0.5 (E0.5). Stage matching was based on limb bud developmental stage [63], since limb stage and nerve invasion are correlated [4,64].

### Retrograde and anterograde labeling of limb and ventral flank nerves

For retrograde labeling, horseradish peroxidase (HRP; 20% (w/v) in 1% lysolecithin/PBS) or tetramethylrhodamine-coupled, lysinated dextran (RDA; 50% (w/v) MW 3,000; Molecular Probes, Carlsbad, CA, USA) was injected into dorsal limb, ventral limb or ventral flank mesenchyme of explanted E13.5 embryos cultured in oxygenated (95% O_2_, 5% CO_2_) DMEM/F12 media (Invitrogen, Carlsbad, CA, USA) at 30–35°C [11,65]. After 4–6 h tissue was fixed and cryosectioned transversely. Control sections were examined by HRP or RDA and neurofilament (NF) coimmunostaining, and evaluated at the dorsoventral branch point for targeting accuracy. Only embryos with a single labeled nerve branch were analyzed further. Ventral flank tracer injection was confirmed at caudal lumbar axial levels that lacked labeled spinal cord neurons by the presence of labeled dorsal root ganglion neurons.

For anterograde labeling, explanted E12.5 embryos were dorsally laminectomized, and a pulled glass needle was introduced from a dorsomedial aspect towards the LMC. Approximately 10–40 nl (empirically determined to label specifically the most medial LMC neurons) of HRP or RDA was injected into every second lumbar spinal cord segment. *Hb9-GFP*^*Tg *^transgene [46] GFP fluorescence at E12.5 is weaker in medial than in lateral LMC neurons, and some injections were guided under UV illumination to this GFP^*low *^region. Labeling accuracy was determined in cryosections co-immunostained for HRP or RDA and NF or LMC division markers. Of 67 injected embryos, 10 (5 normal, 5 mutant) were further analyzed, because the vast majority of HRP+ cells were medial LMC neurons. In some embryos a few labeled axons appeared to be from sensory or medial MMC neurons.

### Immunostaining, ephrin detection and *in situ *hybridization

Antibody stains on cryosections were performed following standard methods [9,11]. Primary antibodies, dilutions and sources: rabbit anti-EphA4 1:1,000 (Zymed, Carlsbad, CA, USA); goat anti-HRP 1:1,000 (Jackson ImmunoResearch Labs, West Grove, PA, USA); mouse anti-Lim3 (61-8C10) 1:50, mouse anti-NF (2H3) 1:100 and mouse anti-Isl1/2 (4D5) 1:100 (Developmental Studies Hybridoma Bank, Iowa City, IA, USA); rabbit anti-Lim1/2 1:4,000, guinea pig anti-Lim3 1:4000, rabbit anti-Isl1/2 (K4) 1:2,500, guinea pig anti-Isl1/2 1:16,000, guinea pig anti-murine Lmx1b 1:16,000, guinea pig anti-FoxP1 1:500 and guinea pig anti-Scip 1:4,000 [9,12,44]. Secondary antibody conjugates, all raised in donkey: Alexa-488 1:2,000 (Molecular Probes), Cy3 1:2,000 or Cy5 1:1,000 (Jackson ImmunoResearch Labs). EphA4-AP fusion protein [66] was used to detect ephrin-A protein in limb cryosections [12].

Non-radioactive RNA *in situ *hybridization on cryosectioned mouse tissue was performed as described [67]. Probes used: mouse *Plzf *[68] and mouse *Lmx1b *[40].

### Quantification

Neurons retrogradely labeled by HRP or RDA and colabeled with LMC markers were counted [33]. Four or more sections from three or more embryos encompassing at least 150, and typically more than 250, retrogradely labeled cells were counted for each condition and marker combination. Statistical comparisons were performed using a two-tailed heteroscedastic Student's *t*-test, with a significance limit of P < 0.05. Values are reported as mean ± standard error of the mean (SEM).

### Imaging and morphometric analysis

Expression patterns were compared using multicolor immunofluorescence imaging or overlaying of RNA *in situ *hybridization or EphA4-AP staining patterns from adjacent cryosections [12]. For *Lim1*^*tlz *^nerves, immunofluorescence intensity of NF+ and LacZ+ nerve branches were compared using the ImageJ program as described [11,23]. The rostrocaudal extent of motor columns, pools and nerve branches was determined by scoring alternate transverse sections for marker presence. The length of the region was then calculated by multiplying the number of sections between the first and last positive section by the section thickness. Typically >80% of internal sections were positive for a given nerve branch.

## Competing interests

The author(s) declare that they have no competing interests.

## Authors' contributions

VL executed most of the experiments, and participated in experimental design, data interpretation and manuscript preparation. EL conceived of the study, performed the experiments in Figure 7, and participated in experimental design, data interpretation and manuscript preparation. Both authors read and approved the final manuscript.

## Supplementary Material

Additional file 1 Motor axons that project to the ventral flank express multiple pool markers. Spinal cords of normal E13.5 embryos retrogradely labeled from the hindlimb ventral flank were immunostained for colocalization of tracer, the medial LMC marker Isl1 and the motor pool markers Nkx6.1 and Er81. Neurons colabeling with either or neither pool marker were detected. **(a) **Schematic of the retrograde labeling experiment. **(b) **Representative image showing colocalization of tracer, Nkx6.1 and Isl1 (white arrowheads). **(c) **Representative image showing colocalization of tracer, Nkx6.1 and Isl1 (white arrowhead) and tracer and Isl1, but not Nkx6.1 (yellow arrows). **(d) **Representative image showing colocalization of tracer, Er81 and Isl1 (white arrowhead) and tracer and Isl1, but not Er81 (yellow arrows). Click here for file

Additional file 2 BmprIaflox/- mutant forelimb mesenchyme has normal dorsoventral polarity with respect to the dorsoventral motor axon projection decision. Forelimb markers were defined and used to assess the character of *BmprIa*^*flox*/- ^mutant forelimbs, while *Tbx4 *and *Tbx5 *were found not to be useful general limb markers. **(a-c) **Upper panels: comparison of marker expression patterns in control limbs at E11, prior to NF+ axon entry into hindlimb and just after NF+ axons entry into forelimb mesenchyme (n = 8 embryos). (a) In hindlimbs *Plzf *marks general limb mesenchyme, and while *Tbx4 *is also expressed in hindlimb mesenchyme, its proximal ventral boundary extends into ventral flank mesenchyme. (b) As in hindlimbs (see Figure 2) *Plzf *is a general forelimb mesenchyme marker, while Lmx1b marks dorsal forelimb. (c) Comparison of *Tbx5 *with *Plzf *and Lmx1b in forelimb mesenchyme demonstrates that its anterior proximal expression boundary is distal to that of *Plzf *and expression extends into ventral flank mesenchyme. *Tbx4 *and *Tbx5 *are therefore not useful as general limb mesenchyme markers with respect to the dorsoventral choice point, but do mark ventral flank mesenchyme. Lower panels (a): comparative expression of *Plzf *and *Tbx5 *(n = 8 embryos) in stage-matched *Brn4-cre*^*Tg*/-^, *BmprIa*^*flox*/- ^mutant hindlimbs. *Tbx5 *expression is retained in the mutant ventral flank tissue, consistent with maintenance of a ventral flank identity. Lower panels (b,c): comparative expression of *Plzf*, Lmx1b and *Tbx5 *(n = 8 embryos) in stage-matched *Brn4-cre*^*Tg*/-^, *BmprIa*^*flox*/- ^mutant forelimbs. As in the control limbs, Lmx1b is restricted to the dorsal *Plzf*+ mesenchyme, consistent with normal dorsoventral polarity in the *BmprIa*^*flox*/- ^mutant forelimbs [31]. Additional *in situ *hybridization probes: mouse *Tbx4 *[69] from Naiche Adler and Virginia Papaioannou (Columbia University) and mouse *Tbx5 *[70] from Malcolm Logan (NIMR, London, UK). Several candidate general limb markers were excluded. This was either because the expression domain extended into the flank (*Tbx4 *in hindlimb, [69,71]; *Tbx5 *in forelimb [70,71], the proximal expression boundary was proximal to that of Lmx1b (*Meis1*, *Meis2 *[72]) or the proximal expression boundary was distal to that of Lmx1b (*Zic2*, *Lhx2*, *Lhx9 *[73-75]) (n = 8 embryos). Click here for file

Additional file 3 Lateral LMC projections are normal in BmprIaflox/- forelimbs. Forelimb axonal projection patterns were examined in control and mutant embryos using a *Lim1*^*tlz *^allele to label axons of Lim1+ lateral LMC neurons with tau-LacZ. **(a,c) **Nerves have started invading both normal (a) and mutant (c) forelimbs at E11.75. NF+ nerves (yellow) branch at the base of the limb and a dorsal branch invades Lmx1b+ dorsal limb (blue) and a ventral branch invades Lmx1b- ventral limb. The ventral branch bifurcates distal to the plexus into ventral limb and ventral flank branches. Boxed areas are shown at higher magnification in (b,d). **(b,d) **LacZ+ lateral LMC axons are readily detected only in the dorsal nerve branch of both normal (b) and mutant (d) embryos. **(e) **LacZ immunoreactivity was quantified and normalized for neurofilament immunoreactivity in each nerve branch, with the ventral limb and flank branches measured distal to their separation. Values are presented in relative units that represent the proportion of LacZ signal in each branch. Relative signal in normal limbs (white boxes), dorsal: ventral: flank, mean ± SEM: 4.7 ± 0.5: 0.90 ± 0.03: 1 ± 0.1, n = 6 embryos. Relative signal in mutant limbs (black boxes): 4.2 ± 1.5: 0.80 ± 0.1: 1 ± 0.15, n = 6 embryos; P = 0.90 (D versus D), 0.65 (V versus V), 0.44 (F versus F), two-tailed *t*-test. D, dorsal limb branch; V, ventral limb branch; F, ventral flank branch. Arrows point to LacZ+ nerves. Dotted lines in (a,c) mark areas magnified in (b,d). n = 10 embryos in (a-d). Click here for file

Additional file 4 No stalled axons are detected at the DV choice point in BmprIaflox/-. The presynaptic marker VAChT stains primarily the distal portion of the NF+ axons, and was used to look for growth cone accumulation at the dorsoventral choice point. **(a) **Diagram of the DV choice point. Boxed area corresponds to images in (b,c). At **(b) **both forelimb and **(c) **hindlimb level, in normal and mutant embryos, VAChT is detected at similarly low levels at the dorsoventral choice point, but stronger distally, indicating that axons are not stalled in the mutants. Embryos are at E11.75, after the axons have started invading the limb. Nerve branches: D, dorsal; V, ventral; F, flank. Arrows: nerve plexus. n = 5 embryos. The expression of VAChT and several additional putative growth cone markers was assessed in mouse at E11.0–12.5. Although the markers used (actin, VAMP2, hamartin, ERM) are all expressed in mouse growth cones in vitro after E14.5, at E11.5–E12.5 none was found to stain exclusively growth cones (not shown). Only VAChT was differentially distributed to the distal part of the axons composing limb nerves. Antibodies used and their concentrations: rabbit anti-hamartin (HF3, HF6) 1:200, provided by Vijaya Ramesh (Massachusetts General Hospital, Boston); mouse IgM anti-ERM (13H9) 1:100 provided by Frank Solomon (MIT, Cambridge, MA); Alexa-488 phalloidin 1:100 (Molecular Probes); from Urs Rutishauser (Sloan-Kettering Institute, New York). Rabbit anti-synaptophysin 1:500 (Zymed); goat anti-vesicular acetylcholine transporter (VAChT) (Zymed) 1:1,000; mouse anti-VAMP2/synaptobrevin 1:1,000 (Synaptic Systems) [76]. Click here for file

Additional file 5 The limb level spinal cord of BmprIaflox/- embryos is normal. While the overall patterning of the spinal cord in *BmprIa*^*flox*/- ^embryos has been reported to be normal [77], the spinal cord motor neurons were not examined in detail. We therefore asked whether brachial and lumbar ventral spinal cord neuronal populations are generated and patterned normally in *BmprIa*^*flox*/- ^embryos. We compared the expression of motor neuron and ventral interneuron progenitor markers [1,78,79] in *BmprIa*^*flox*/- ^mutant and normal sibling embryos at E10.0. We also compared the developing postmitotic motor neuron populations from E10.0 through E13.5 using markers [1,9] for medial MMC (Lim3+ Isl1+ Lim1-), medial LMC (Lim3- Isl1+ Lim1-) and lateral LMC (Lim3- Isl1- Lim1+) motor neurons. We observed no significant difference between *BmprIa*^*flox*/- ^mutant and normal sibling progenitor or motor neuron populations at either axial level. These data thus provide molecular evidence that the limb level spinal cord motor neuron and ventral interneuron populations are generated normally in *BmprIa*^*flox*/- ^mutant embryos. Molecular markers of spinal cord neuronal populations were used to assess the patterning and development of the *BmprIa*^*flox*/- ^mutant ventral spinal cord. **(a) **Within the ventricular spinal cord of normal and *BmprIa*^*flox*/- ^mutant embryos at E10 there is no difference in the presence or relative positions of progenitor populations for V3 interneurons (Nkx6.1+ Nkx2.2+ Olig2-), motor neurons (Nkx6.1+ Nkx2.2- Olig2+), and V2 interneurons (Nkx6.1+ Nkx2.2- Olig2-). **(b) **At E10 early postmitotic populations of medial MMC (Isl1+ Lim3+) and medial LMC (Isl1+ Lim3-) motor neurons are similar between normal and mutant. Later-born lateral LMC (Lim1+ Isl1-) neurons are not detectable at this time. **(c) **At E11.5 there is no difference in the lateral LMC (Isl1- Lim1+) and medial LMC plus medial MMC (Isl1+ Lim1-) populations of normal and mutant. Lateral LMC neuron cell bodies are still migrating toward their final lateral destination. Additional motor neuron marker antibodies used and their concentrations: rabbit anti-Nkx6.1 1:4,000, guinea pig anti-Nkx6.1 1:8,000, mouse anti-Nkx2.2 (75-5A5) 1:50, rabbit anti-Nkx2.2 1:4,000, rabbit anti-Olig2 1:16,000, guinea pig anti-Olig2 1:8,000, provided by Susan Morton and Thomas Jessell (Columbia University) [78,80]. Click here for file

Additional file 6 The motor innervation of BmprIaflox/- forelimbs is normal. Retrograde labeling of nerves from **(a,b,e) **dorsal or **(c,d,f) **ventral limb mesenchyme at E13.5 marks cell bodies of spinal cord neurons that contribute to each nerve branch. (a-d) upper panels are control embryos, lower panels *BmprIa*^*flox*/- ^mutants. (a) Dorsal nerves originate from lateral LMC in both normal and mutant as only Lim1+ Isl1- lateral LMC cells are HRP+. (b) Diagram of the experiment in (a). (c) Ventral nerve originates from Lim1- Isl1+ HRP+ medial LMC in both normal and mutant embryos. (d) Diagram of the experiment in (c). Quantification of (e) dorsal and (f) ventral retrograde labeling data. (e) The percentage of dorsally-labeled lateral or medial LMC cells does not differ significantly between normal and mutant (normal: white bar, lateral LMC 93% ± 2%, n = 3 embryos, N > 110 HRP+ neurons; mutant: black bar, lateral LMC 95% ± 3%, n = 4 embryos, N > 110 HRP+ neurons; *P *= 0.67). (f) The percentage of ventrally-labeled lateral or medial LMC cells does not differ significantly between normal and mutant (normal: white bar, medial LMC 93% ± 3%; n = 6 embryos, N > 150 HRP+ neurons; mutant: black bar, medial LMC 93% ± 1%; n = 6 embryos, N > 300 HRP+ neurons; *P *= 0.83). None of the retrogradely labeled neurons express the medial MMC marker Lim3, indicating the limb nerve branches are composed of LMC neurons (n = 3 embryos for normal and mutant embryos; data not shown). (a,c) Dotted lines: lateral and medial LMC outlines. (b,d) Boxed areas: regions imaged in (a,c). (e,f) LMC(l): lateral LMC, LMC(m): medial LMC, white boxes: normal embryos, black boxes: mutant embryos, mean ± SEM. Lateral LMC cells: Lim1+ Isl1- HRP+. Medial LMC cells: Lim1- Isl1+ HRP+. Arrowheads: representative HRP+ Isl1+ or HRP+ Lim1+ neurons. Click here for file

Additional file 7 The lumbar spinal cord of Hb9cre/+, BmprIaflox/- embryos is normal. **(a) **At E11.5 medial MMC (Isl1+ Lim3+), lateral LMC (Lim1+ Isl1-) and medial LMC (Isl1+ Lim1-) motor neuron populations are similar between normal and mutant. Lateral LMC neurons are still migrating toward their final lateral destination and are thus intermingled with medial LMC neurons. **(b) **Cre protein immunoreactivity is present in a majority of Isl1+ motor neurons in both *Hb9*^*cre*/+ ^positive controls and *Hb9*^*cre*/+^, *BmprIa*^*flox*/- ^mutants. Click here for file

## References

[B1] Jessell TM (2000). Neuronal specification in the spinal cord: inductive signals and transcriptional codes. Nat Rev Genet.

[B2] Landmesser LT (2001). The acquisition of motoneuron subtype identity and motor circuit formation. Int J Dev Neurosci.

[B3] Shirasaki R, Pfaff SL (2002). Transcriptional codes and the control of neuronal identity. Annu Rev Neurosci.

[B4] Lance-Jones C, Landmesser L (1981). Pathway selection by embryonic chick motoneurons in an experimentally altered environment. Proc R Soc Lond B Biol Sci.

[B5] Tosney KW, Landmesser LT (1985). Specificity of early motoneuron growth cone outgrowth in the chick embryo. J Neurosci.

[B6] Romanes GJ (1964). The Motor Pools of the Spinal Cord. Prog Brain Res.

[B7] McHanwell S, Biscoe TJ (1981). The localization of motoneurons supplying the hindlimb muscles of the mouse. Philos Trans R Soc Lond B Biol Sci.

[B8] Hollyday M, Jacobson RD (1990). Location of motor pools innervating chick wing. Journal of Comparative Neurology.

[B9] Tsuchida T, Ensini M, Morton SB, Baldassare M, Edlund T, Jessell TM, Pfaff SL (1994). Topographic organization of embryonic motor neurons defined by expression of LIM homeobox genes. Cell.

[B10] Sharma K, Sheng HZ, Lettieri K, Li H, Karavanov A, Potter S, Westphal H, Pfaff SL (1998). LIM homeodomain factors Lhx3 and Lhx4 assign subtype identities for motor neurons. Cell.

[B11] Kania A, Johnson RL, Jessell TM (2000). Coordinate roles for LIM homeobox genes in directing the dorsoventral trajectory of motor axons in the vertebrate limb. Cell.

[B12] Kania A, Jessell TM (2003). Topographic motor projections in the limb imposed by LIM homeodomain protein regulation of ephrin-A:EphA interactions. Neuron.

[B13] Shirasaki R, Lewcock JW, Lettieri K, Pfaff SL (2006). FGF as a target-derived chemoattractant for developing motor axons genetically programmed by the LIM code. Neuron.

[B14] Ferguson BA (1983). Development of motor innervation of the chick following dorsal-ventral limb bud rotations. J Neurosci.

[B15] Whitelaw V, Hollyday M (1983). Neural pathway constraints in the motor innervation of the chick hindlimb following dorsoventral rotations of distal limb segments. J Neurosci.

[B16] Tosney KW, Landmesser LT (1984). Pattern and specificity of axonal outgrowth following varying degrees of chick limb bud ablation. J Neurosci.

[B17] Lance-Jones CC (1986). Motoneuron projection patterns in chick embryonic limbs with a double complement of dorsal thigh musculature. Dev Biol.

[B18] Eberhart J, Barr J, O'Connell S, Flagg A, Swartz ME, Cramer KS, Tosney KW, Pasquale EB, Krull CE (2004). Ephrin-A5 exerts positive or inhibitory effects on distinct subsets of EphA4-positive motor neurons. J Neurosci.

[B19] Huber AB, Kania A, Tran TS, Gu C, De Marco Garcia N, Lieberam I, Johnson D, Jessell TM, Ginty DD, Kolodkin AL (2005). Distinct roles for secreted semaphorin signaling in spinal motor axon guidance. Neuron.

[B20] Kramer ER, Knott L, Su F, Dessaud E, Krull CE, Helmbacher F, Klein R (2006). Cooperation between GDNF/Ret and ephrinA/EphA4 Signals for Motor-Axon Pathway Selection in the Limb. Neuron.

[B21] Helmbacher F, Schneider-Maunoury S, Topilko P, Tiret L, Charnay P (2000). Targeting of the EphA4 tyrosine kinase receptor affects dorsal/ventral pathfinding of limb motor axons. Development.

[B22] Eberhart J, Swartz ME, Koblar SA, Pasquale EB, Krull CE (2002). EphA4 Constitutes a Population-Specific Guidance Cue for Motor Neurons. Dev Biol.

[B23] Hanson MG, Landmesser LT (2004). Normal patterns of spontaneous activity are required for correct motor axon guidance and the expression of specific guidance molecules. Neuron.

[B24] Landmesser L (1978). The development of motor projection patterns in the chick hind limb. J Physiol.

[B25] Tosney KW, Landmesser LT (1985). Development of the major pathways for neurite outgrowth in the chick hindlimb. Dev Biol.

[B26] Hollyday M (1990). Specificity of initial axonal projections to embryonic chick wing. Journal of Comparative Neurology.

[B27] Ohta K, Iwamasa H, Drescher U, Terasaki H, Tanaka H (1997). The inhibitory effect on neurite outgrowth of motoneurons exerted by the ligands ELF-1 and RAGS. Mech Dev.

[B28] Eberhart J, Swartz M, Koblar SA, Pasquale EB, Tanaka H, Krull CE (2000). Expression of EphA4, ephrin-A2 and ephrin-A5 during axon outgrowth to the hindlimb indicates potential roles in pathfinding. Dev Neurosci.

[B29] Altman J, Bayer S (1995). Atlas of prenatal brain development.

[B30] April EW (1997). Clinical Anatomy. The National Medical Series for Independent Study.

[B31] Ahn K, Mishina Y, Hanks MC, Behringer RR, Crenshaw EB (2001). BMPR-IA signaling is required for the formation of the apical ectodermal ridge and dorsal-ventral patterning of the limb. Development.

[B32] Tamura S, Morikawa Y, Iwanishi H, Hisaoka T, Senba E (2003). Expression pattern of the winged-helix/forkhead transcription factor Foxp1 in the developing central nervous system. Gene Expr Patterns.

[B33] Lin JH, Saito T, Anderson DJ, Lance-Jones C, Jessell TM, Arber S (1998). Functionally related motor neuron pool and muscle sensory afferent subtypes defined by coordinate ETS gene expression. Cell.

[B34] Sander M, Paydar S, Ericson J, Briscoe J, Berber E, German M, Jessell TM, Rubenstein JL (2000). Ventral neural patterning by Nkx homeobox genes: Nkx6.1 controls somatic motor neuron and ventral interneuron fates. Genes Dev.

[B35] Heydemann A, Nguyen LC, Crenshaw EB (2001). Regulatory regions from the Brn4 promoter direct LACZ expression to the developing forebrain and neural tube. Brain Res Dev Brain Res.

[B36] Soshnikova N, Zechner D, Huelsken J, Mishina Y, Behringer RR, Taketo MM, Crenshaw EB, Birchmeier W (2003). Genetic interaction between Wnt/beta-catenin and BMP receptor signaling during formation of the AER and the dorsal-ventral axis in the limb. Genes Dev.

[B37] Ferns MJ, Hollyday M (1993). Motor innervation of dorsoventrally reversed wings in chick/quail chimeric embryos. J Neurosci.

[B38] Barna M, Pandolfi PP, Niswander L (2005). Gli3 and Plzf cooperate in proximal limb patterning at early stages of limb development. Nature.

[B39] Riddle RD, Ensini M, Nelson C, Tsuchida T, Jessell TM, Tabin C (1995). Induction of the LIM homeobox gene Lmx1 by WNT7a establishes dorsoventral pattern in the vertebrate limb. Cell.

[B40] Chen H, Lun Y, Ovchinnikov D, Kokubo H, Oberg KC, Pepicelli CV, Gan L, Lee B, Johnson RL (1998). Limb and kidney defects in Lmx1b mutant mice suggest an involvement of LMX1B in human nail patella syndrome. Nat Genet.

[B41] Iwamasa H, Ohta K, Yamada T, Ushijima K, Terasaki H, Tanaka H (1999). Expression of Eph receptor tyrosine kinases and their ligands in chick embryonic motor neurons and hindlimb muscles. Dev Growth Differ.

[B42] Flanagan JG, Vanderhaeghen P (1998). The ephrins and Eph receptors in neural development. Annu Rev Neurosci.

[B43] Monuki ES, Weinmaster G, Kuhn R, Lemke G (1989). SCIP: a glial POU domain gene regulated by cyclic AMP. Neuron.

[B44] Dasen JS, Tice BC, Brenner-Morton S, Jessell TM (2005). A Hox regulatory network establishes motor neuron pool identity and target-muscle connectivity. Cell.

[B45] Wilson JM, Rempel J, Brownstone RM (2004). Postnatal development of cholinergic synapses on mouse spinal motoneurons. J Comp Neurol.

[B46] Wichterle H, Lieberam I, Porter JA, Jessell TM (2002). Directed differentiation of embryonic stem cells into motor neurons. Cell.

[B47] Yang X, Arber S, William C, Li L, Tanabe Y, Jessell TM, Birchmeier C, Burden SJ (2001). Patterning of muscle acetylcholine receptor gene expression in the absence of motor innervation. Neuron.

[B48] Nagy JI, Senba E (1985). Neural relations of cremaster motoneurons, spinal cord systems and the genitofemoral nerve in the rat. Brain Res Bull.

[B49] Zempoalteca R, Martinez-Gomez M, Hudson R, Cruz Y, Lucio RA (2002). An anatomical and electrophysiological study of the genitofemoral nerve and some of its targets in the male rat. J Anat.

[B50] Calguner E, Erdogan D, Elmas C, Bahcelioglu M, Gozil R, Ayhan MS (2006). Innervation of the rat anterior abdominal wall as shown by modified Sihler's stain. Med Princ Pract.

[B51] Gerrits PO, Boers J, Holstege G (1997). The lumbar cord location of the motoneurons innervating psoas and iliacus muscles: a single and double labeling study in the female Syrian golden hamster. Neurosci Lett.

[B52] Nicolopoulos-Stournaras S, Iles JF (1983). Motor neuron columns in the lumbar spinal cord of the rat. J Comp Neurol.

[B53] Tani M, Kida MY, Akita K (1994). Relationship between the arrangement of motoneuron pools in the ventral horn and ramification pattern of the spinal nerve innervating trunk muscles in the cat (Felis domestica). Exp Neurol.

[B54] Ertekin C, Bademkiran F, Yildiz N, Ozdedeli K, Altay B, Aydogdu I, Uludag B (2005). Central and peripheral motor conduction to cremasteric muscle. Muscle Nerve.

[B55] Turney BW, Rowan-Hull AM, Brown JM (2003). The innervation of FGF-induced additional limbs in the chick embryo. J Anat.

[B56] Sockanathan S, Jessell TM (1998). Motor neuron-derived retinoid signaling specifies the subtype identity of spinal motor neurons. Cell.

[B57] Haase G, Dessaud E, Garces A, de Bovis B, Birling M, Filippi P, Schmalbruch H, Arber S, deLapeyriere O (2002). GDNF acts through PEA3 to regulate cell body positioning and muscle innervation of specific motor neuron pools. Neuron.

[B58] Livet J, Sigrist M, Stroebel S, De Paola V, Price SR, Henderson CE, Jessell TM, Arber S (2002). ETS gene Pea3 controls the central position and terminal arborization of specific motor neuron pools. Neuron.

[B59] Liu JP, Laufer E, Jessell TM (2001). Assigning the positional identity of spinal motor neurons: rostrocaudal patterning of Hox-c expression by FGFs, Gdf11, and retinoids. Neuron.

[B60] Nelsen OE (1953). Comparative Embryology of the Vertebrates.

[B61] Mishina Y, Suzuki A, Ueno N, Behringer RR (1995). Bmpr encodes a type I bone morphogenetic protein receptor that is essential for gastrulation during mouse embryogenesis. Genes Dev.

[B62] Mishina Y, Hanks MC, Miura S, Tallquist MD, Behringer RR (2002). Generation of Bmpr/Alk3 conditional knockout mice. Genesis.

[B63] Wanek N, Muneoka K, Holler-Dinsmore G, Burton R, Bryant SV (1989). A staging system for mouse limb development. Journal of Experimental Zoology.

[B64] Wang G, Scott SA (2000). The "waiting period" of sensory and motor axons in early chick hindlimb: its role in axon pathfinding and neuronal maturation. J Neurosci.

[B65] Lance-Jones C, Landmesser L (1980). Motoneurone projection patterns in the chick hind limb following early partial reversals of the spinal cord. J Physiol.

[B66] Feldheim DA, Kim YI, Bergemann AD, Frisen J, Barbacid M, Flanagan JG (2000). Genetic analysis of ephrin-A2 and ephrin-A5 shows their requirement in multiple aspects of retinocollicular mapping. Neuron.

[B67] Laufer E, Dahn R, Orozco OE, Yeo CY, Pisenti J, Henrique D, Abbott UK, Fallon JF, Tabin C (1997). Expression of Radical fringe in limb-bud ectoderm regulates apical ectodermal ridge formation. Nature.

[B68] Avantaggiato V, Pandolfi PP, Ruthardt M, Hawe N, Acampora D, Pelicci PG, Simeone A (1995). Developmental analysis of murine Promyelocyte Leukemia Zinc Finger (PLZF) gene expression: implications for the neuromeric model of the forebrain organization. J Neurosci.

[B69] Naiche LA, Papaioannou VE (2003). Loss of Tbx4 blocks hindlimb development and affects vascularization and fusion of the allantois. Development.

[B70] Rallis C, Bruneau BG, Del Buono J, Seidman CE, Seidman JG, Nissim S, Tabin CJ, Logan MP (2003). Tbx5 is required for forelimb bud formation and continued outgrowth. Development.

[B71] Gibson-Brown JJ, Agulnik SI, Chapman DL, Alexiou M, Garvey N, Silver LM, Papaioannou VE (1996). Evidence of a role for T-box genes in the evolution of limb morphogenesis and the specification of forelimb/hindlimb identity. Mech Dev.

[B72] Mercader N, Leonardo E, Azpiazu N, Serrano A, Morata G, Martinez C, Torres M (1999). Conserved regulation of proximodistal limb axis development by Meis1/Hth. Nature.

[B73] Nagai T, Aruga J, Takada S, Gunther T, Sporle R, Schughart K, Mikoshiba K (1997). The expression of the mouse Zic1, Zic2, and Zic3 gene suggests an essential role for Zic genes in body pattern formation. Dev Biol.

[B74] Rincon-Limas DE, Lu CH, Canal I, Calleja M, Rodriguez-Esteban C, Izpisua-Belmonte JC, Botas J (1999). Conservation of the expression and function of apterous orthologs in *Drosophila* and mammals. Proc Natl Acad Sci U S A.

[B75] Retaux S, Rogard M, Bach I, Failli V, Besson MJ (1999). Lhx9: a novel LIM-homeodomain gene expressed in the developing forebrain. J Neurosci.

[B76] Ramesh V (2004). Merlin and the ERM proteins in Schwann cells, neurons and growth cones. Nat Rev Neurosci.

[B77] Wine-Lee L, Ahn KJ, Richardson RD, Mishina Y, Lyons KM, Crenshaw EB (2004). Signaling through BMP type 1 receptors is required for development of interneuron cell types in the dorsal spinal cord. Development.

[B78] Novitch BG, Chen AI, Jessell TM (2001). Coordinate regulation of motor neuron subtype identity and pan-neuronal properties by the bHLH repressor Olig2. Neuron.

[B79] McMahon AP (2000). Neural patterning: the role of Nkx genes in the ventral spinal cord. Genes Dev.

[B80] Briscoe J, Pierani A, Jessell TM, Ericson J (2000). A homeodomain protein code specifies progenitor cell identity and neuronal fate in the ventral neural tube. Cell.

