# Supplementary figures and images for: Lateral motor column axons execute a ternary trajectory choice between limb and body tissues
Source: Neural Dev. 2007 Jul 2;2:13. doi: 10.1186/1749-8104-2-13 (PMC1949814; doi:10.1186/1749-8104-2-13)

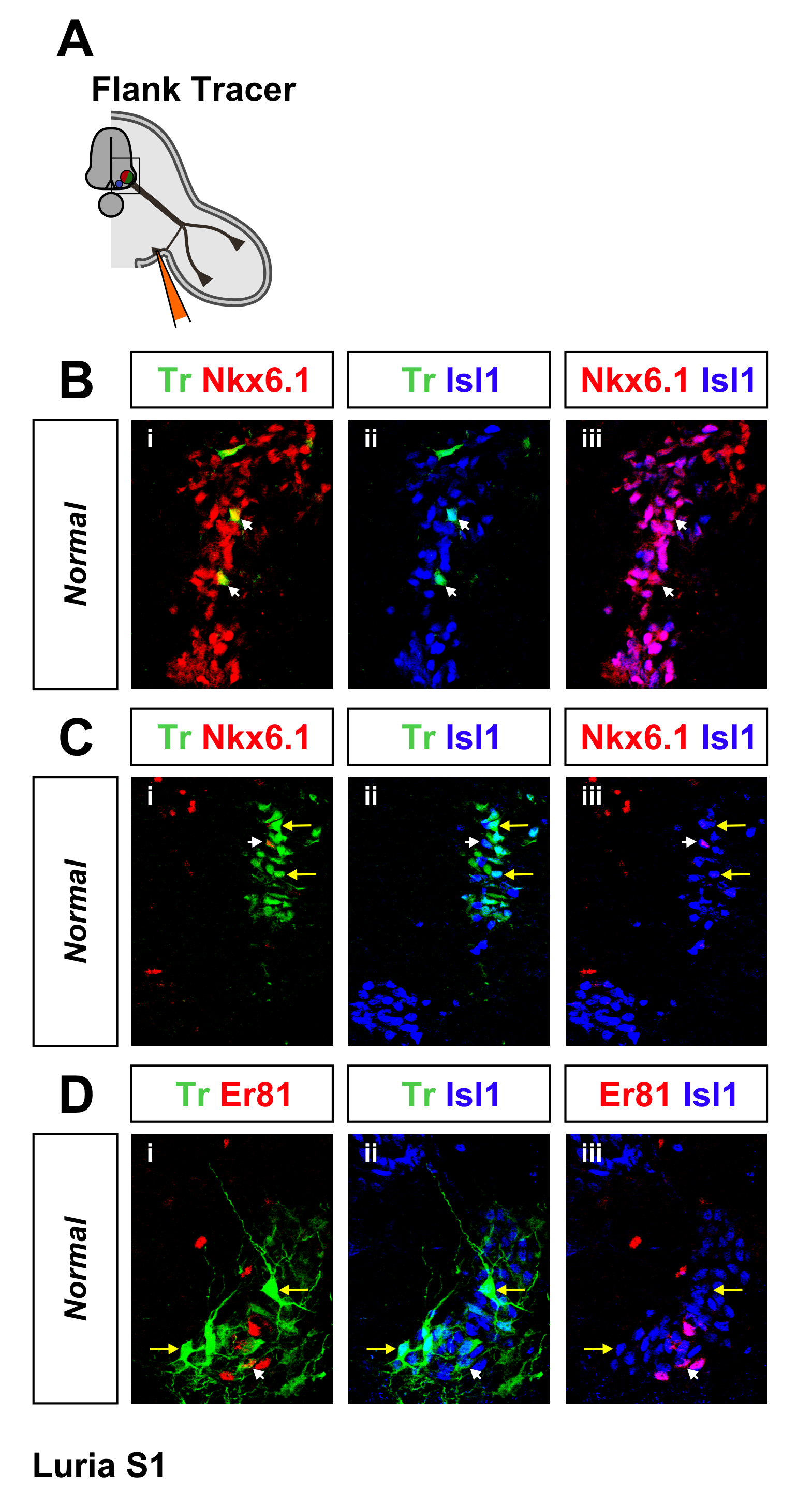

Supplement: Additional file 1 — Motor axons that project to the ventral flank express multiple pool markers. Spinal cords of normal E13.5 embryos retrogradely labeled from the hindlimb ventral flank were immunostained for colocalization of tracer, the medial LMC marker Isl1 and the motor pool markers Nkx6.1 and Er81. Neurons colabeling with either or neither pool marker were detected. (a) Schematic of the retrograde labeling experiment. (b) Representative image showing colocalization of tracer, Nkx6.1 and Isl1 (white arrowheads). (c) Representative image showing colocalization of tracer, Nkx6.1 and Isl1 (white arrowhead) and tracer and Isl1, but not Nkx6.1 (yellow arrows). (d) Representative image showing colocalization of tracer, Er81 and Isl1 (white arrowhead) and tracer and Isl1, but not Er81 (yellow arrows). [file 1749-8104-2-13-S1.png]

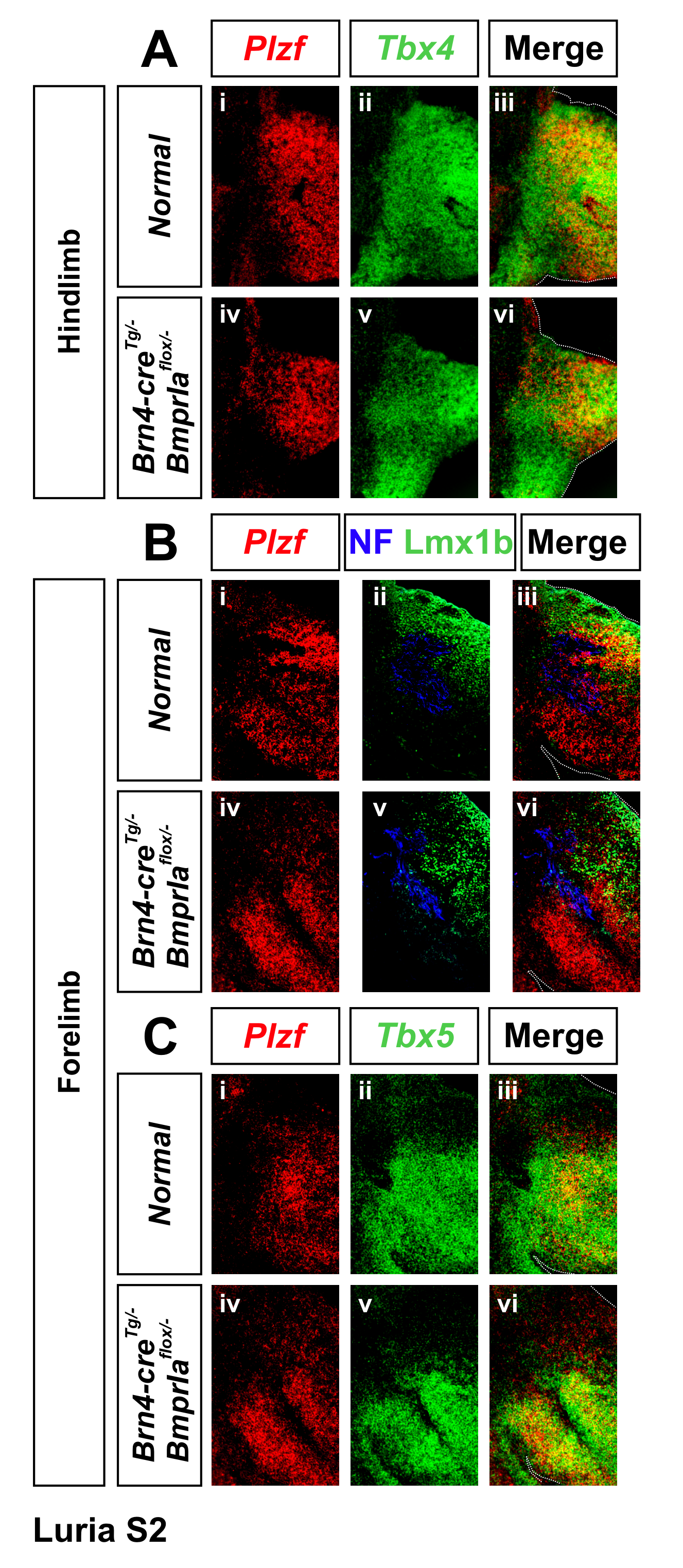

Supplement: Additional file 2 — BmprIaflox/- mutant forelimb mesenchyme has normal dorsoventral polarity with respect to the dorsoventral motor axon projection decision. Forelimb markers were defined and used to assess the character of BmprIaflox/- mutant forelimbs, while Tbx4 and Tbx5 were found not to be useful general limb markers. (a-c) Upper panels: comparison of marker expression patterns in control limbs at E11, prior to NF+ axon entry into hindlimb and just after NF+ axons entry into forelimb mesenchyme (n = 8 embryos). (a) In hindlimbs Plzf marks general limb mesenchyme, and while Tbx4 is also expressed in hindlimb mesenchyme, its proximal ventral boundary extends into ventral flank mesenchyme. (b) As in hindlimbs (see Figure 2) Plzf is a general forelimb mesenchyme marker, while Lmx1b marks dorsal forelimb. (c) Comparison of Tbx5 with Plzf and Lmx1b in forelimb mesenchyme demonstrates that its anterior proximal expression boundary is distal to that of Plzf and expression extends into ventral flank mesenchyme. Tbx4 and Tbx5 are therefore not useful as general limb mesenchyme markers with respect to the dorsoventral choice point, but do mark ventral flank mesenchyme. Lower panels (a): comparative expression of Plzf and Tbx5 (n = 8 embryos) in stage-matched Brn4-creTg/-, BmprIaflox/- mutant hindlimbs. Tbx5 expression is retained in the mutant ventral flank tissue, consistent with maintenance of a ventral flank identity. Lower panels (b,c): comparative expression of Plzf, Lmx1b and Tbx5 (n = 8 embryos) in stage-matched Brn4-creTg/-, BmprIaflox/- mutant forelimbs. As in the control limbs, Lmx1b is restricted to the dorsal Plzf+ mesenchyme, consistent with normal dorsoventral polarity in the BmprIaflox/- mutant forelimbs [31]. Additional in situ hybridization probes: mouse Tbx4 [69] from Naiche Adler and Virginia Papaioannou (Columbia University) and mouse Tbx5 [70] from Malcolm Logan (NIMR, London, UK). Several candidate general limb markers were excluded. This was either because the expressi [file 1749-8104-2-13-S2.png]

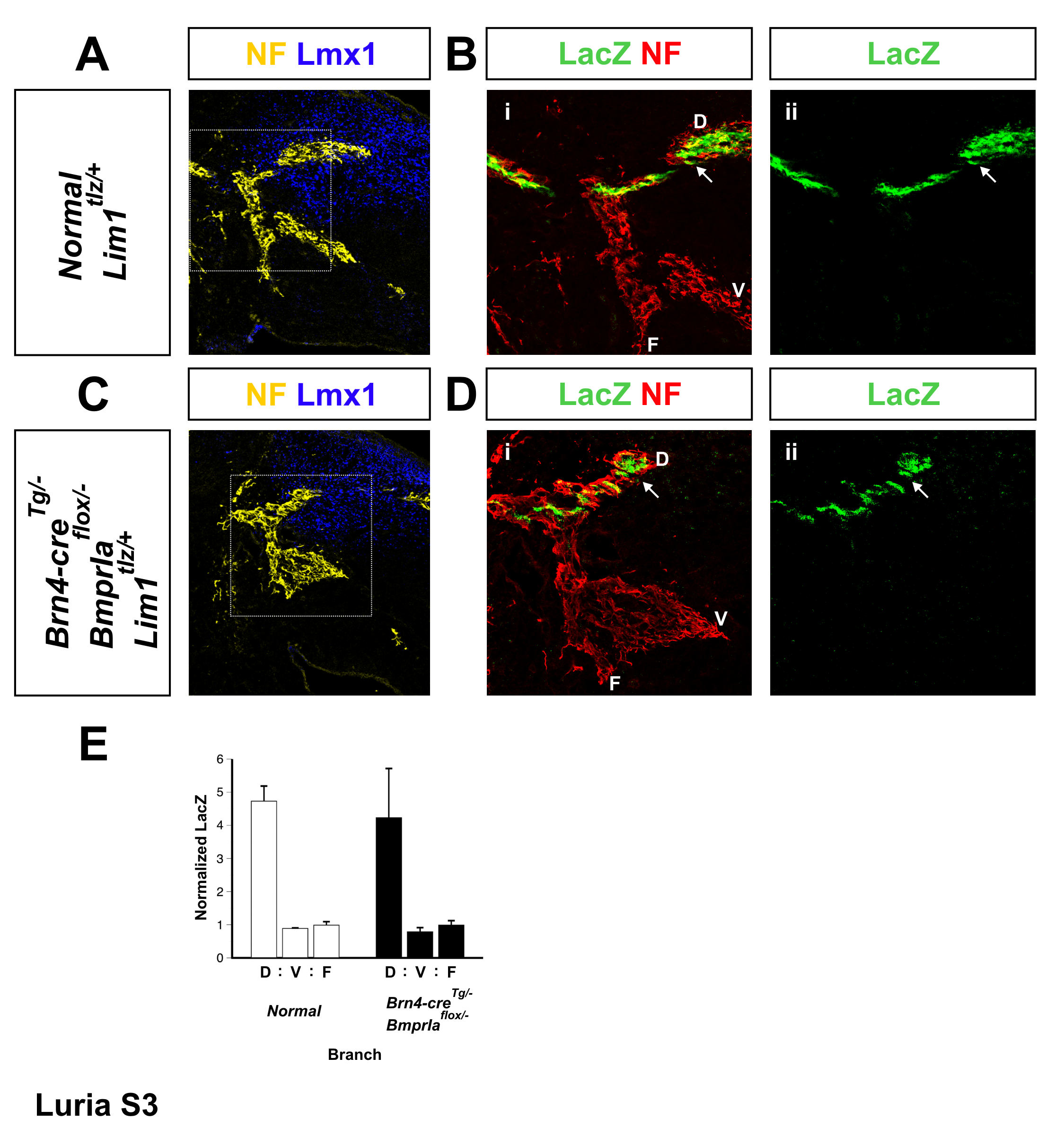

Supplement: Additional file 3 — Lateral LMC projections are normal in BmprIaflox/- forelimbs. Forelimb axonal projection patterns were examined in control and mutant embryos using a Lim1tlz allele to label axons of Lim1+ lateral LMC neurons with tau-LacZ. (a,c) Nerves have started invading both normal (a) and mutant (c) forelimbs at E11.75. NF+ nerves (yellow) branch at the base of the limb and a dorsal branch invades Lmx1b+ dorsal limb (blue) and a ventral branch invades Lmx1b- ventral limb. The ventral branch bifurcates distal to the plexus into ventral limb and ventral flank branches. Boxed areas are shown at higher magnification in (b,d). (b,d) LacZ+ lateral LMC axons are readily detected only in the dorsal nerve branch of both normal (b) and mutant (d) embryos. (e) LacZ immunoreactivity was quantified and normalized for neurofilament immunoreactivity in each nerve branch, with the ventral limb and flank branches measured distal to their separation. Values are presented in relative units that represent the proportion of LacZ signal in each branch. Relative signal in normal limbs (white boxes), dorsal: ventral: flank, mean ± SEM: 4.7 ± 0.5: 0.90 ± 0.03: 1 ± 0.1, n = 6 embryos. Relative signal in mutant limbs (black boxes): 4.2 ± 1.5: 0.80 ± 0.1: 1 ± 0.15, n = 6 embryos; P = 0.90 (D versus D), 0.65 (V versus V), 0.44 (F versus F), two-tailed t-test. D, dorsal limb branch; V, ventral limb branch; F, ventral flank branch. Arrows point to LacZ+ nerves. Dotted lines in (a,c) mark areas magnified in (b,d). n = 10 embryos in (a-d). [file 1749-8104-2-13-S3.png]

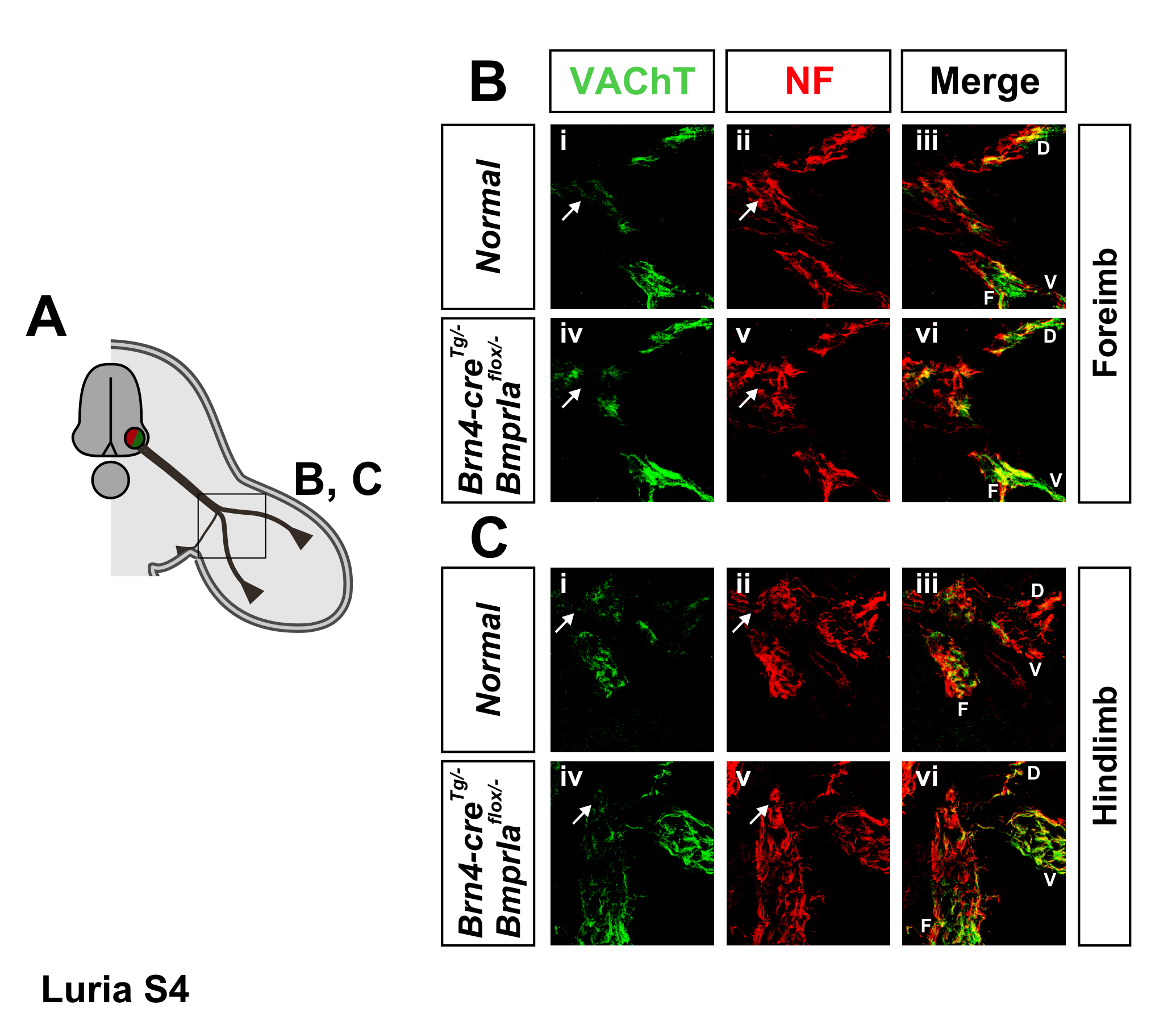

Supplement: Additional file 4 — No stalled axons are detected at the DV choice point in BmprIaflox/-. The presynaptic marker VAChT stains primarily the distal portion of the NF+ axons, and was used to look for growth cone accumulation at the dorsoventral choice point. (a) Diagram of the DV choice point. Boxed area corresponds to images in (b,c). At (b) both forelimb and (c) hindlimb level, in normal and mutant embryos, VAChT is detected at similarly low levels at the dorsoventral choice point, but stronger distally, indicating that axons are not stalled in the mutants. Embryos are at E11.75, after the axons have started invading the limb. Nerve branches: D, dorsal; V, ventral; F, flank. Arrows: nerve plexus. n = 5 embryos. The expression of VAChT and several additional putative growth cone markers was assessed in mouse at E11.0–12.5. Although the markers used (actin, VAMP2, hamartin, ERM) are all expressed in mouse growth cones in vitro after E14.5, at E11.5–E12.5 none was found to stain exclusively growth cones (not shown). Only VAChT was differentially distributed to the distal part of the axons composing limb nerves. Antibodies used and their concentrations: rabbit anti-hamartin (HF3, HF6) 1:200, provided by Vijaya Ramesh (Massachusetts General Hospital, Boston); mouse IgM anti-ERM (13H9) 1:100 provided by Frank Solomon (MIT, Cambridge, MA); Alexa-488 phalloidin 1:100 (Molecular Probes); from Urs Rutishauser (Sloan-Kettering Institute, New York). Rabbit anti-synaptophysin 1:500 (Zymed); goat anti-vesicular acetylcholine transporter (VAChT) (Zymed) 1:1,000; mouse anti-VAMP2/synaptobrevin 1:1,000 (Synaptic Systems) [76]. [file 1749-8104-2-13-S4.png]

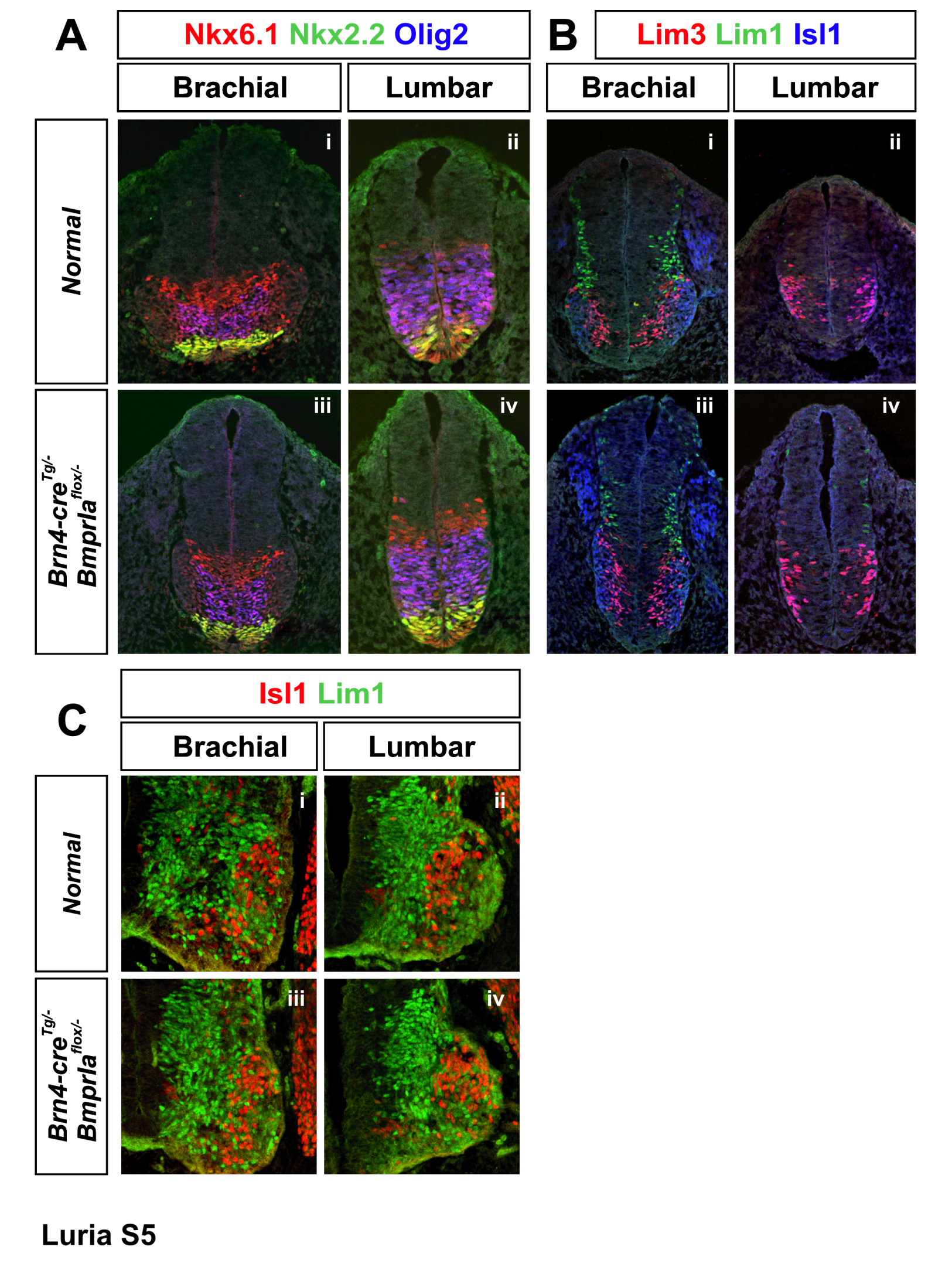

Supplement: Additional file 5 — The limb level spinal cord of BmprIaflox/- embryos is normal. While the overall patterning of the spinal cord in BmprIaflox/- embryos has been reported to be normal [77], the spinal cord motor neurons were not examined in detail. We therefore asked whether brachial and lumbar ventral spinal cord neuronal populations are generated and patterned normally in BmprIaflox/- embryos. We compared the expression of motor neuron and ventral interneuron progenitor markers [1,78,79] in BmprIaflox/- mutant and normal sibling embryos at E10.0. We also compared the developing postmitotic motor neuron populations from E10.0 through E13.5 using markers [1,9] for medial MMC (Lim3+ Isl1+ Lim1-), medial LMC (Lim3- Isl1+ Lim1-) and lateral LMC (Lim3- Isl1- Lim1+) motor neurons. We observed no significant difference between BmprIaflox/- mutant and normal sibling progenitor or motor neuron populations at either axial level. These data thus provide molecular evidence that the limb level spinal cord motor neuron and ventral interneuron populations are generated normally in BmprIaflox/- mutant embryos. Molecular markers of spinal cord neuronal populations were used to assess the patterning and development of the BmprIaflox/- mutant ventral spinal cord. (a) Within the ventricular spinal cord of normal and BmprIaflox/- mutant embryos at E10 there is no difference in the presence or relative positions of progenitor populations for V3 interneurons (Nkx6.1+ Nkx2.2+ Olig2-), motor neurons (Nkx6.1+ Nkx2.2- Olig2+), and V2 interneurons (Nkx6.1+ Nkx2.2- Olig2-). (b) At E10 early postmitotic populations of medial MMC (Isl1+ Lim3+) and medial LMC (Isl1+ Lim3-) motor neurons are similar between normal and mutant. Later-born lateral LMC (Lim1+ Isl1-) neurons are not detectable at this time. (c) At E11.5 there is no difference in the lateral LMC (Isl1- Lim1+) and medial LMC plus medial MMC (Isl1+ Lim1-) populations of normal and mutant. Lateral LMC neuron cell bodies are still migrating toward their final [file 1749-8104-2-13-S5.png]

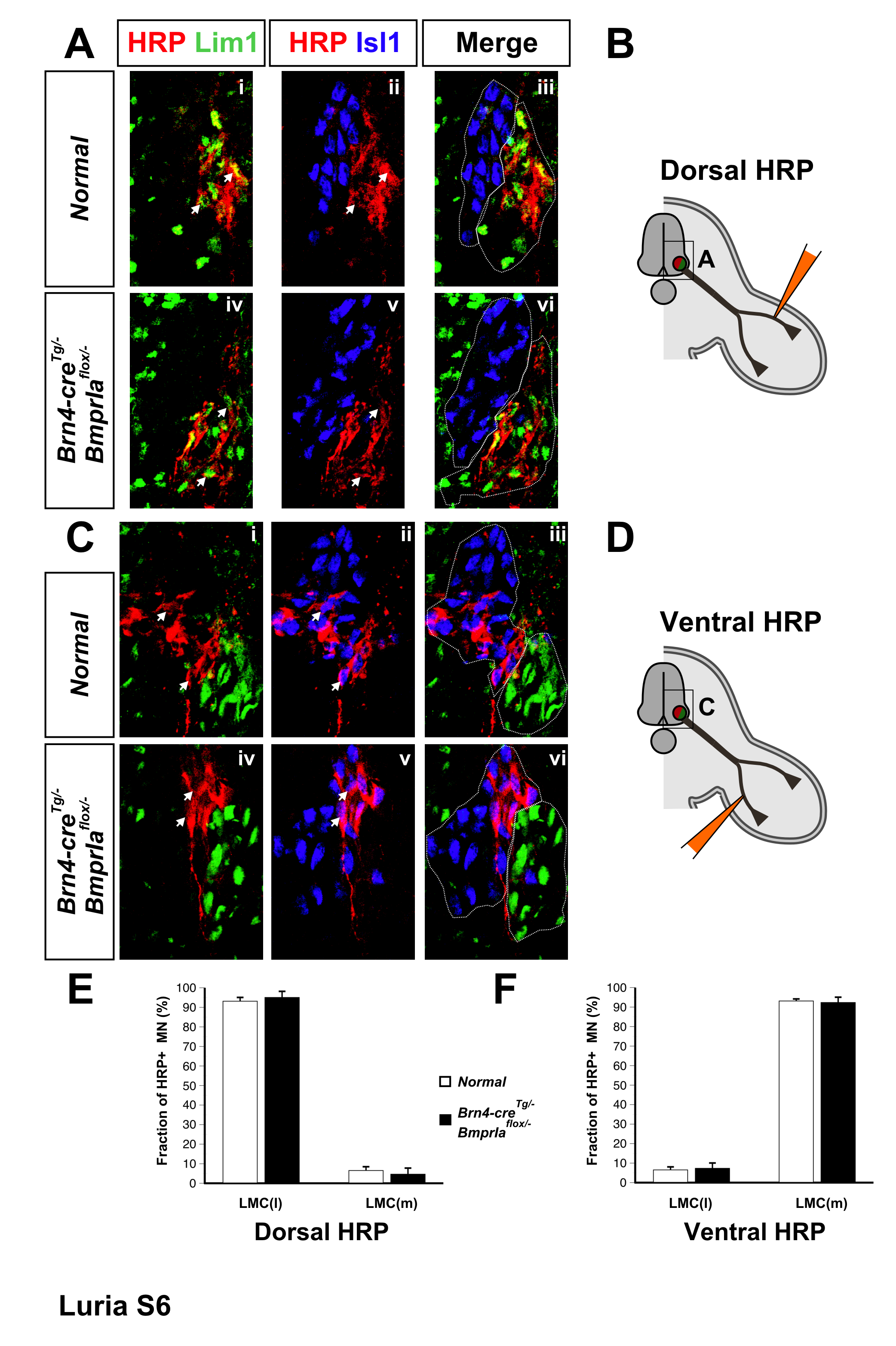

Supplement: Additional file 6 — The motor innervation of BmprIaflox/- forelimbs is normal. Retrograde labeling of nerves from (a,b,e) dorsal or (c,d,f) ventral limb mesenchyme at E13.5 marks cell bodies of spinal cord neurons that contribute to each nerve branch. (a-d) upper panels are control embryos, lower panels BmprIaflox/- mutants. (a) Dorsal nerves originate from lateral LMC in both normal and mutant as only Lim1+ Isl1- lateral LMC cells are HRP+. (b) Diagram of the experiment in (a). (c) Ventral nerve originates from Lim1- Isl1+ HRP+ medial LMC in both normal and mutant embryos. (d) Diagram of the experiment in (c). Quantification of (e) dorsal and (f) ventral retrograde labeling data. (e) The percentage of dorsally-labeled lateral or medial LMC cells does not differ significantly between normal and mutant (normal: white bar, lateral LMC 93% ± 2%, n = 3 embryos, N > 110 HRP+ neurons; mutant: black bar, lateral LMC 95% ± 3%, n = 4 embryos, N > 110 HRP+ neurons; P = 0.67). (f) The percentage of ventrally-labeled lateral or medial LMC cells does not differ significantly between normal and mutant (normal: white bar, medial LMC 93% ± 3%; n = 6 embryos, N > 150 HRP+ neurons; mutant: black bar, medial LMC 93% ± 1%; n = 6 embryos, N > 300 HRP+ neurons; P = 0.83). None of the retrogradely labeled neurons express the medial MMC marker Lim3, indicating the limb nerve branches are composed of LMC neurons (n = 3 embryos for normal and mutant embryos; data not shown). (a,c) Dotted lines: lateral and medial LMC outlines. (b,d) Boxed areas: regions imaged in (a,c). (e,f) LMC(l): lateral LMC, LMC(m): medial LMC, white boxes: normal embryos, black boxes: mutant embryos, mean ± SEM. Lateral LMC cells: Lim1+ Isl1- HRP+. Medial LMC cells: Lim1- Isl1+ HRP+. Arrowheads: representative HRP+ Isl1+ or HRP+ Lim1+ neurons. [file 1749-8104-2-13-S6.png]

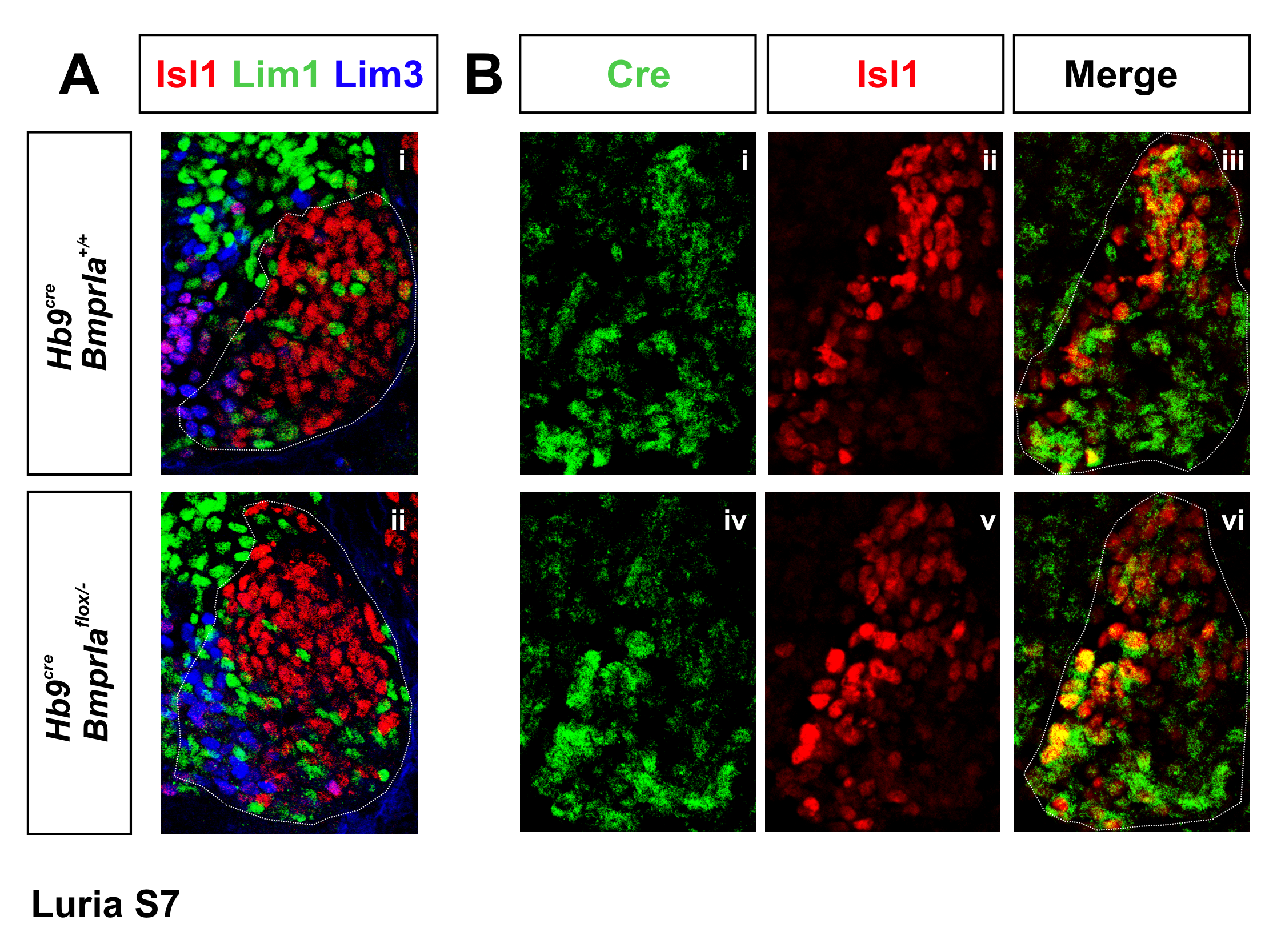

Supplement: Additional file 7 — The lumbar spinal cord of Hb9cre/+, BmprIaflox/- embryos is normal. (a) At E11.5 medial MMC (Isl1+ Lim3+), lateral LMC (Lim1+ Isl1-) and medial LMC (Isl1+ Lim1-) motor neuron populations are similar between normal and mutant. Lateral LMC neurons are still migrating toward their final lateral destination and are thus intermingled with medial LMC neurons. (b) Cre protein immunoreactivity is present in a majority of Isl1+ motor neurons in both Hb9cre/+ positive controls and Hb9cre/+, BmprIaflox/- mutants. [file 1749-8104-2-13-S7.png]
